# DNA Barcoding of *Rhodiola* (Crassulaceae): A Case Study on a Group of Recently Diversified Medicinal Plants from the Qinghai-Tibetan Plateau

**DOI:** 10.1371/journal.pone.0119921

**Published:** 2015-03-16

**Authors:** Jian-Qiang Zhang, Shi-Yong Meng, Jun Wen, Guang-Yuan Rao

**Affiliations:** 1 College of Life Sciences, Peking University, Beijing, China; 2 Department of Botany, National Museum of Natural History, MRC 166, Smithsonian Institution, Washington, D.C., United States of America; Chinese Academy of Medical Sciences, Peking Union Medical College, CHINA

## Abstract

DNA barcoding, the identification of species using one or a few short standardized DNA sequences, is an important complement to traditional taxonomy. However, there are particular challenges for barcoding plants, especially for species with complex evolutionary histories. We herein evaluated the utility of five candidate sequences — *rbcL*, *matK*, *trnH-psbA*, *trnL-F* and the internal transcribed spacer (ITS) — for barcoding *Rhodiola* species, a group of high-altitude plants frequently used as adaptogens, hemostatics and tonics in traditional Tibetan medicine. *Rhodiola* was suggested to have diversified rapidly recently. The genus is thus a good model for testing DNA barcoding strategies for recently diversified medicinal plants. This study analyzed 189 accessions, representing 47 of the 55 recognized *Rhodiola* species in the *Flora of China* treatment. Based on intraspecific and interspecific divergence and degree of monophyly statistics, ITS was the best single-locus barcode, resolving 66% of the *Rhodiola* species. The core combination *rbcL*+*matK* resolved only 40.4% of them. Unsurprisingly, the combined use of all five loci provided the highest discrimination power, resolving 80.9% of the species. However, this is weaker than the discrimination power generally reported in barcoding studies of other plant taxa. The observed complications may be due to the recent diversification, incomplete lineage sorting and reticulate evolution of the genus. These processes are common features of numerous plant groups in the high-altitude regions of the Qinghai-Tibetan Plateau.

## Introduction

DNA barcoding refers to rapid, accurate taxon identification using one or a few short, standardized DNA region(s) [1,2]. Through large-scale standardized sequencing of the mitochondrial gene CO1, it has become an efficient tool for identifying species in many animal groups [2]. However, three obstacles still hinder its extensive application in plants, despite strenuous efforts [3–5]. Firstly, designing universal primers for targeted markers in all plants is problematic. Secondly, rates of successful amplification and sequencing of candidate DNA markers widely vary amongst plant groups. Thirdly, between-species differences in candidate barcodes are also highly variable and even non-existent in some taxa. Nevertheless, DNA barcoding can often be applied in plants by using two or three DNA markers [4,6,7].

An effective barcoding marker must be easy to amplify and sequence using a universal pair of primers, suitably long, usually 500–800 bp [5], and sufficiently variable between species but homogeneous within a species to distinguish closely related species robustly [3]. Several DNA regions have been identified as potentially suitable barcodes. The plastid gene *rbcL* and the nuclear ribosomal internal transcribed spacer (ITS) can reportedly distinguish species of *Moraea* Mill. and *Protea* L., according to BLAST tests [8]. Kress and colleagues have recommended the use of ITS and plastid *trnH-psbA* sequences, and subsequently *trnH-psbA* and *rbcL* genes, as two-locus universal barcodes for land plants [4]. The CBOL Plant Working Group recommended the combination rbcL + matK as a core plant barcode, and also advocated the plastid trnH-psbA and ITS [9] as complementary markers.

*Rhodiola* L. (Crassulaceae) consists of about 70 species mainly distributed in high altitudes and cold regions of the Northern Hemisphere [10,11]. There are about 55 *Rhodiola* species recognized in China (16 species endemic), especially in the western alpine regions (i.e., the Hengduan Mountains and the Qinghai-Tibetan Plateau) [10]. Species of this genus are herbaceous perennials that often grow on gravel-covered slopes or in cracks of exposed rocks at ca. 3500–5000 m elevations, thus collecting and studying them have been notoriously difficult [10]. *Rhodiola* species, historically used as adaptogens in Russia and northern Europe, have been widely recognized for enhancing human resistance to stress or fatigue and promoting longevity [12–14]. In China, the *Rhodiola* species known as Hongjingtian have been frequently used as adaptogens, hemostatics, and tonics in traditional Tibetan medicines for thousands of years [14]. The type species *R*. *rosea* L. is a popular traditional medicinal plant in east Europe and Asia, with a reputation for stimulating the nervous system, decreasing depression, enhancing work performance, eliminating fatigue, and preventing high-altitude sickness [15]. The roots and rhizomes of *R*. *crenulata* (J. D. Hooker & Thomson) H. Ohba have been included in the *Pharmacopoeia of China* [16]. Furthermore, several other *Rhodiola* species, such as *R*. *sachalinensis* A. Bor., *R*. *himalensis* (D. Dons) S. H. Fu, *R*. *serrata* H. Ohba, and *R*. *fastigiata* (Hook. f. et Thomson) S. H. Fu are also used as medicines in China. Consequently, many species of this genus are severely endangered in Asia due to excessive and indiscriminate exploitation [17,18]. In spite of their wide use and medicinal importance, the identification of closely related species of *Rhodiola* is often difficult due to their morphological similarity.

A recent phylogenetic study of *Rhodiola* revealed significant convergent evolution of important morphological characters, such as dioecy and marcescent flowering stems [19]. Consequently, many of the previously defined infrageneric taxa are not monophyletic. Historical biogeographic studies have suggested that rapid radiations occurred in the evolution of this genus [20]. *Rhodiola* is thus an excellent model for evaluating the effectiveness and universality of rbcL + matK as a core plant barcode, and the plastid trnH-psbA and ITS as complementary markers in a group of closely related, recently diversified plant species. Furthermore, although several studies have assessed the suitability of barcoding markers for identifying important medicinal plants [21,22], they have generally focused on a small fraction of species in a genus, or single targeted species. Few studies have tested the suitability of DNA barcodes using extensive samples covering most species of a medicinal plant genus of moderate size. In the present study, we analyzed a broad set of samples of *Rhodiola* as a model group of recently diversified medicinal plants, to evaluate the proposed core and complementary DNA barcodes, as well as *trnL-F*. We also specifically assessed the power of the barcodes to discriminate six widely used medicinal species of *Rhodiola*.

## Materials and Methods

### Ethics statement

No specific permits were required for the described locations in China because all researchers collecting the samples had introduction letters from College of Life Sciences, Peking University, Beijing. The field studies did not involve protected species. The localities of all accessions sampled were shown in S1 Table.

### Plant materials

In total, 189 accessions representing 47 *Rhodiola* species (including the widely used medicinal species) were collected from sites in Xizang, Qinghai, Gansu, Sichuan, Yunnan, and Xinjiang provinces of China, and north America from 2009 to 2012 (see S1 Table for collection information and NCBI accession numbers). Fresh leaves were dried in silica gel upon collection. Three to six accessions per taxon were sampled to cover the diversity within each taxon and most of their respective geographical ranges. Voucher specimens of the collected taxa were deposited in the Herbarium of Peking University (PEY) and the National Herbarium of the United States of America (US).

### DNA extraction, amplification, and sequencing

Genomic DNA was isolated from ca. 15 mg of each leaf sample following the CTAB protocol [23]. The primers for amplification and sequencing were: ITS-1 and ITS-4 for ITS [11], psbAF and trnHR for *trnH-psbA* [24], c and f for *trnL-F* [25], rbcL-1F and rbcL-R for the *rbcL* gene [26], KIM 3-F and KIM 1-R for *matK* [27] (Table 1). The four candidate DNA barcodes were amplified by the polymerase chain reaction (PCR) in 20 μL mixtures containing 2 μL of 10 × buffer, 0.5 μM of each primer, 200 μM of each dNTP, 1 U of Taq polymerase (TianGen Biotech, Beijing, China), and 1 μL template genomic DNA. The temperature program consisted of 5 min at 95°C, 35 cycles of 1 min at 95°C, 1 min at 56°C, and 1 min at 72°C, with a final extension of 5 min at 72°C. PCR products were purified by polyethylene glycol (PEG) precipitation [28], then sequenced using BigDye 3.1 reagents with an ABI 3730 automated sequencer (Applied Biosystems, Foster City, California) at the Biomed Corporation (Beijing, China).

**Table 1:** PCR primers and references used in the present study.

Region	Name of primer	Primer sequence 5’-3’	References
*rbcL*	rbcL-1F	ATGTCACCACAAACAGAAAC	[26]
	rbcL-R	TCACAAGCAGCTAGTTCAGGACTC	
*matK*	KIM 3-F	CGTACAGTACTTTTGTGTTTACGAG	[27]
	KIM 1-R	ACCCAGTCCATCTGGAAATCTTGGTTC	
*trnH-psbA*	psbAF	GTTATGCATGAACGTAATGCTC	[24]
	trnHR	CGCGCATGGTGGATTCACAAATC	
*trnL-F*	c	CGAAATCGGTAGACGCTACG	[25]
	f	ATTTGAACTGGTGACACGAG	
ITS	ITS-1	TCCGTAGGTGAACCTGCGG	[11]
	ITS-4	TCCTCCGCTTATTGATATGC	

ITS = internal transcript spacer.

### Data analyses

Contigs were assembled and edited using the ContigExpress module of Vector NTI Suite 6.0 (InforMax). Sequences were aligned using MUSCLE 3.8.31 [29], followed by manual adjustments in Geneious 7.1.7 [30]. We calculated the Kimura 2-parameter (K2P) distance for all five DNA regions in MEGA v. 6.0 [31] to estimate intra- and inter-specific divergence, and assessed its significance using the median and Wilcox two-sample tests (http://www.fon.hum.uva.nl/Service/Statistics.html). We also graphed the distribution of intra- and inter-specific divergence (i.e., K2P distances) of each candidate barcode. Here intra-specific distances include all possible intra-specific comparisons and inter-specific distances represent all possible inter-specific comparisons. We also used the BLAST procedure to evaluate the generic-level identification power of the five tested markers, using every sequence generated for the five candidate barcodes as a seed sequence to check whether best matches in the National Centre for Biotechnology Information nucleotide database (http://www.ncbi.nlm.nih.gov/BLAST/) were from the same genus.

To evaluate the monophyly of the individuals representing the same species based on morphological assessment, tree-based methods were used to display the molecular identification results. The species identification rates of the barcodes (either singly or in all combinations) were determined by evaluating the percentages of their assignments for each species that were monophyletic according to the UPGMA, NJ, MP, and ML analyses. The neighbor-joining (NJ) and the unweighted pair group method with arithmetic mean (UPGMA) trees were reconstructed using MEGA v. 6.0 with the K2P model [31]. The program PAUP v. 4.0b10 [32] was used to generate the maximum parsimony (MP) tree with a heuristic search strategy followed by random addition starting trees with tree-bisection-reconnection (TBR) branch swapping and MulTrees selected. Indels were treated as missing data and all characters were weighted equally. Support for individual nodes was assessed by calculating bootstrap values [33]. Parsimony bootstrap (PB) values were obtained from 1,000 replicates of heuristic searches as described above (TBR branch swapping and MulTrees selected), but with branch swapping limited to 10 million rearrangements per replicate due to memory constraints. Nucleotide substitution model parameters were determined using the Akaike Information Criterion (AIC) in Modeltest version 3.7 [34,35]. Maximum likelihood (ML) analyses were performed using RAxML 8.0.0 with 1000 bootstraps under the GTRIG model [36]. We used the following code to set the parameters: –b 1000 –m GTR –v e –f e –t e –a e –o tlr (see the RAxML manual for details).

We also used the genetic distance-based program TaxonDNA to analyze the species identification rates of the DNA barcodes using three criteria: “Best Match” (which assigns queries to species with the best-matching sequences, regardless of their similarity); “Best Close Match” (which assigns queries to species if a threshold similarity is met); and “All Species Barcodes” (which assigns queries to species if they match all known barcodes for the species and there are at least two conspecific matches) [37].

## Results

### Amplification and sequencing

The primers for all five selected DNA regions were found to be applicable for all the 47 species of *Rhodiola*, and 933 barcode sequences were generated from the study. Newly generated sequences were deposited in the GenBank database with accession numbers shown in S1 Table. The amplification and bidirectional sequencing success rates consistently exceeded 95% for all the markers except *matK* (Table 2). We tested the amplification efficiency of several other published primers for amplifying *matK*, including a pair designed for amplifying Saxifragaceae sequences [38], and a recently designed pair of “universal” primers for an angiosperm barcode [39], but the success rate was low. The primer pair used in the present study (KIM 3-F and KIM 1-R [27]) worked the best, but the success rate for *matK* was still lower than that of the other markers (Table 2).

**Table 2:** Information used to evaluate the utility of the five DNA barcoding loci.

	*rbcL*	*matK*	*trnH-psbA*	*trnL-F*	ITS
Universal primer	YES	YES	YES	YES	YES
PCR success (%)	95	88	100	100	98
Sequencing success (%)	100	96	100	100	96
Aligned length (%)	1415	847	423	927	668
# of informative sites/variable sites	68/172	62/101	77/103	51/119	204/254
# of individuals	192	168	194	192	187
No. of SNPs	172	101	80	72	214
No. of indels	0	0	23	47	40

### Alignment, variability, and BLAST procedure

The aligned lengths of the *rbcL*, *matK*, *trnH-psbA*, *trnL-F* and ITS barcode data set were 1415, 847, 423, 927, and 668 bp, respectively. Length variation exists in each region, which is 1259–1272 bp for *rbcL*, 762–786 bp for *matK*, 277–347 bp for *trnH-psbA*, and 600–642 bp for ITS. The numbers of informative sites and variable sites (Table 2) were the highest for ITS (204 and 254, respectively), and the lowest for *matK* (62 and 101, respectively). The best matches to sequences of the five candidate barcodes from the 47 investigated species, identified by BLAST searches of the NCBI database, were all from species of *Rhodiola*.

### Monophyly tests of species based on phylogenetic trees

In the monophyly tests based on phylogenetic trees, UPGMA analyses provided the highest indications of discriminatory power, followed by NJ, ML and MP analyses. As single barcodes, ITS provided the highest species identification rate (66.0%), followed by *matK*, *trnH-psbA*, *trnL-F* and *rbcL* (36.2, 36.2, 29.8 and 19.1%, respectively). Furthermore, even all four plastid-derived barcodes provided a lower identification rate (61.7%) than ITS, demonstrating the discriminatory power of ITS as a DNA barcode. However, the highest percentage (80.9%) was achieved using all five candidate barcodes, followed by combinations including ITS (e.g., 76.6% using both *rbcL + matK + trnL-F +* ITS and *rbcL + matK + trnH-psbA +* ITS). The “core” barcode combination *rbcL* and *matK* yielded a species identification rate of just 40.4% (Table 3).

**Table 3:** Percentage of *Rhodiola* species recovered as monophyletic based on phylogenetic trees for each barcode.

Potential barcode	UPGMA	NJ	MP	ML
*rbcL*	19.1 (14.9)	19.1 (14.9)	14.9 (12.8)	17.0 (14.9)
*matK*	36.2 (34.0)	36.2 (34.0)	31.9 (29.8)	34.0 (31.9)
*trnH-psbA*	36.2 (31.9)	34.0 (31.9)	31.9 (29.8)	34.0 (31.9)
*trnL-F*	29.8 (21.3)	29.8 (21.3)	23.4 (19.1)	27.7 (21.3)
ITS	66.0 (63.8)	66.0 (63.8)	63.8 (59.6)	66.0 (61.7)
*rbcL*+ *matK*	40.4 (36.2)	38.3 (36.2)	31.9 (21.3)	38.3 (31.9)
*rbcL*+ *trnH-psbA*	42.6 (29.8)	42.6 (29.8)	36.2 (31.9)	40.4 (29.8)
*rbcL*+ trnL-F	31.9 (23.4)	31.9 (23.4)	29.8 (19.1)	29.8 (19.1)
*rbcL*+ ITS	70.2 (63.8)	70.2 (63.8)	63.8 (57.4)	68.1 (63.8)
*matK*+ *trnH-psbA*	48.9 (36.2)	48.9 (36.2)	42.6 (29.8)	46.8 (34.0)
*matK*+ *trnL-F*	48.9 (42.6)	48.9 (42.6)	44.7 (36.2)	46.8 (42.6)
*matK* + ITS	72.3 (70.2)	72.3 (70.2)	61.7 (57.4)	70.2 (61.7)
*trnH-psbA* + ITS	68.1 (61.7)	68.1 (61.7)	61.7 (51.1)	63.8 (61.7)
*trnH-psbA* + trnL-F	31.9 (21.3)	31.9 (21.3)	23.4 (19.1)	27.7 (21.3)
*trnL-F*+ ITS	68.1 (61.7)	68.1 (61.7)	59.6 (51.1)	68.1 (61.7)
*rbcL+ matK+ trnH-psbA*	59.6 (57.4)	57.4 (57.4)	51.1 (42.6)	55.3 (53.2)
*rbcL+ matK+ trnL-F*	51.1 (46.8)	51.1 (46.8)	46.8 (36.2)	48.9 (46.8)
*rbcL+ matK+* ITS	74.5 (70.2)	74.5 (70.2)	63.8 (57.4)	74.5 (70.2)
*rbcL +matK+ trnH-psbA+ trnL-F*	61.7 (57.4)	61.7 (57.4)	55.3 (46.8)	59.6 (57.4)
*rbcL +matK+ trnL-F +* ITS	76.6 (72.3)	76.6 (72.3)	70.2 (63.8)	76.6 (70.2)
*rbcL+ matK +trnH-psbA* + ITS	76.6 (74.5)	76.6 (72.3)	63.8 (57.4)	76.6 (70.2)
*rbcL +matK+ trnH-psbA+ trnL-F+* ITS	80.9 (76.6)	80.9 (76.6)	72.3 (68.1)	78.7 (74.5)

Values outside of the parenthesis represent percentage of species-level monophyly; values in the parenthesis indicate species-level monophyly with bootstrap value ⩾ 70.

### Barcoding gap test

The barcoding gaps between intra- and inter-specific distances assessed by graphing the distribution of variation in K2P genetic distance for *rbcL*, *matK*, *trnH-psbA*, *trnL-F* and ITS were shown in S1 Fig. For each barcode candidate, both the median and Wilcoxon two-sample tests showed that the intra-specific distance was always significantly lower than the inter-specific distance (Table 4).

**Table 4:** Results of the median and the Wilcoxon two-sample tests based on interspecific versus intraspecific Kimura 2-parameter distances for each barcode.

Region	#A	#B	Median	p-value	W	p-value	Mean interspecific distance (range)	Mean intraspecific distance (range)
*rbcL*	15590	341	0.003979	< 2.2e-16	1323469	< 2.2e-16	0.0044 (0–0.0400)	0.0025 (0–0.0300)
*matK*	13249	281	0.009151	< 2.2e-16	454669	< 2.2e-16	0.0099 (0–0.0250)	0.0030 (0–0.0170)
*trnH-psbA*	17321	351	0.028425	4.676e-16	189673	< 2.2e-16	0.0288 (0–0.0723)	0.0138 (0–0.0577)
*trnL-F*	17414	352	0.007547	< 2.2e-16	1151017	< 2.2e-16	0.0082 (0–0.0301)	0.0032 (0–0.0200)
ITS	17228	350	0.040675	< 2.2e-16	846358	< 2.2e-16	0.0400 (0–0.0787)	0.0129 (0–0.0600)

#A – No. of interspecific distances; #B – No. of intraspecific distances; ITS, internal transcribed spacer; W, Wilcoxon two-sample test result.

### TaxonDNA analysis

The results of TaxonDNA analysis using three criteria was shown in Table 5. According to the “Best Match” and “Best Close Match” criteria, ITS, *trnH-psbA*, *trnL-F*, *matK* and *rbcL* provided species identifications for 65.4, 38.9, 34.9, 34.5 and 22.3% of the samples, respectively. Slightly lower percentages, but similar patterns, were obtained using the “All species Barcodes” strategy. ITS alone and combinations including ITS provided higher success rates than other markers (and combinations of markers) and generally combinations provided higher success rates than single markers (Table 5) based on all three criteria.

**Table 5:** Success rates of species identification based on TaxonDNA analysis.

Potential barcode	Best match (%)	Best close match (%)	All sequences barcode (%)
*rbcL*	22.34	22.34	29.04
*matK*	34.54	34.54	23.03
*trnH-psbA*	38.93	38.93	26.32
*trnL-F*	34.92	34.92	25.39
ITS	65.42	65.42	39.36
*rbcL*+ *matK*	34.63	34.63	20.11
*rbcL*+ *trnH-psbA*	46.03	46.03	30.15
*rbcL*+ *trnL-F*	40.21	40.21	25.39
*rbcL*+ ITS	68.32	68.32	36.25
*matK*+ *trnH-psbA*	44.44	44.44	28.57
*matK*+ *trnL-F*	42.32	42.32	19.57
*matK* +ITS	69.67	69.67	37.23
*trnH-psbA* +ITS	74.07	73.54	34.92
*trnH-psbA* + trnL-F	51.85	51.85	26.98
*trnL-F*+ ITS	69.84	69.84	37.03
*rbcL+ matK+ trnH-psbA*	51.85	51.85	29.62
*rbcL+ matK+ trnL-F*	51.85	51.85	23.28
*rbcL+ matK+* ITS	71.56	71.56	38.93
*rbcL +matK+ trnH-psbA+ trnL-F*	53.96	53.96	28.04
*rbcL +matK+ trnL-F +* ITS	71.95	71.95	38.09
*rbcL+ matK +trnH-psbA* + ITS	73.01	73.01	39.68
*rbcL +matK+ trnH-psbA+ trnL-F+* ITS	71.95	71.95	38.09

## Discussion

New techniques are needed to improve descriptive taxonomy and to ensure that organisms used for various scientific and applied purposes are correctly identified [37]. DNA barcoding is still a relatively new technique, and it has been extensively applied for rapidly identifying diverse taxa [21,27]. However, substantial advances are still needed to reliably apply DNA barcoding in plants. There is no consensus on standard markers, procedures and strategies for barcode development, although a “core barcode” and several other combinations of candidate barcode regions have been proposed [5,27]. A standard DNA barcode for plants must be able to differentiate challenging plant species, such as recently diversified taxa with complex evolutionary histories.

### Identification power of barcode loci in *Rhodiola*

As reported in other plant barcoding studies [22,40–42], we also found the ITS region to be the most powerful and the most useful one of the five tested barcodes in *Rhodiola*. ITS, alone or in combination with plastid markers, can discriminate more than 70% of the species in this genus, confirming its utility as a core barcode marker [9]. One of the main constraints of using ITS as a standard barcode is that it is difficult to amplify and sequence in some taxa [40,42], due to incomplete concerted evolution [9]. Although the success rates for ITS amplification and sequencing were high (Table 2), attempts to acquire ITS sequences for some individuals of *R*. *discolor* (Franch.) S. H. Fu and *R*. *bupleuroides* (Wall. ex Hook. f. & Thomson) S. H. Fu failed or were difficult, possibly due to multiple polyploidy events, at least for the *R*. *bupleuroides* samples, with the reported chromosome numbers of this species ranging from 20 to 110 [43].

The plastid markers showed significantly lower discriminatory power than the ITS region, although the CBOL Plant Working Group has recommended *rbcL* and *matK* genes as core plant barcodes [27]. Among the four plastid markers, *rbcL*, which is a coding region, showed the least variability in *Rhodiola* (Tables 2 & 3). Low variability of *rbcL* has also been found in other barcoding studies [40–42]. Thus, this conservative marker is often used to determine the taxonomic affinity of unknown samples. Our BLAST analyses of the *rbcL* sequences all provided best matches with a congeneric species, confirming its utility for placing the plants into the correct genus. Another coding region recommended by the CBOL Plant Working Group is *matK*, which showed lower variability and lower discrimination power than the ITS region, but greater utility than *rbcL* in *Rhodiola* (Tables 2 & 3). However, developing universal primers for *matK* has been reported to be problematic [4,40,44], and we encountered the same obstacle in the present study. Several previously designed primers, including a pair of recently designed “universal primers” for angiosperm barcoding for amplifying partial *matK* regions [39] also failed. The primer pair used in the present study (Table 1) worked relatively well, but the success rate was still lower than that of other barcodes (Table 2).

Compared to the two coding regions (*rbcL*, *matK*), which tend to be more conserved, the two non-coding regions, *trnH-psbA* and *trnL-F*, were more useful for distinguishing similar species. The *trnH-psbA* spacer has been suggested as a robust DNA barcode for various plants, including *Ligustrum* L. [41], orchids [45], and *Tetrastigma* (Miq.) Planch. [40]. Our results show that it has more variable sites, and provided higher species identification rates, than both *rbcL* and *matK* (Tables 2 & 3), even though it is much shorter (423 vs 1415 and 847 bp, Table 2). However, its length may be highly variable, ranging from > 300 bp in some groups [5], such as *Solidago* L., to > 1000 bp in ferns [45], some monocots [46] and conifers [27]. Such length variation may make it difficult for bidirectional sequencing using universal primers as well as for accurate alignment. Our data indicate that the average length of *trnH-psbA* in *Rhodiola* is appropriate for a barcode. We detected five large indels (≥10 bp) in the alignment for this marker, but their occurrence does not seem to correlate with species; for example, the same 14 bp insertion was detected in one *R*. *heterodonta* (Hook. f. & Thomson) Boriss. individual and one *R*. *macrocarpa* (Praeger) S. H. Fu individual. Thus, as pointed out by Kress et al. [5] and Fu et al. [40], indel variations in *trnH-psbA* may not be suitable for distinguishing species.

Few studies have discussed the suitability of *trnL-F* as a potential DNA barcode in angiosperms. It was suggested to be a powerful barcode marker in ferns, because the region shows sufficient variations among species, and the universal primers show high success rates for amplification and sequencing [47,48]. We confirmed the amenability of the *trnL-F* region for amplification and sequencing. Furthermore, although it did not have higher variation (i.e., discrimination power) than *matK* or *trnH*-*psbA*, it was slightly more variable than *rbcL* (Tables 2 & 3). Thus, given the difficulties of amplifying and sequencing *matK* and the potential “indel” problem of the *trnH-psbA* spacer, the *trnL-F* regions seems a promising alternative marker for barcoding *Rhodiola* and other plants.

Multi-locus barcodes have consistently provided stronger identification power than single regions [27]. Accordingly, the combinations of markers generally showed higher discriminatory power than single markers in the present study (Tables 3 & 5). For example, the two core barcodes (*rbcL* and *matK*) in combination distinguished 40.4% of the included *Rhodiola* species, while separately they distinguished 19.1 and 36.2%, respectively. The TaxonDNA analysis based on different criteria showed the same pattern (Table 5). We also tested the robustness of *rbcL* + *matK* + X (ITS, *trnH-psbA*, *trnL-F*, or combinations thereof), as advocated by representatives of the Chinese Plants Barcoding program. The results showed that using more than two cpDNA barcodes increased identification rates, although the ITS region alone seems to discriminate species equally well.

### DNA barcoding evaluation in a well sampled data set

**Fig 1:**
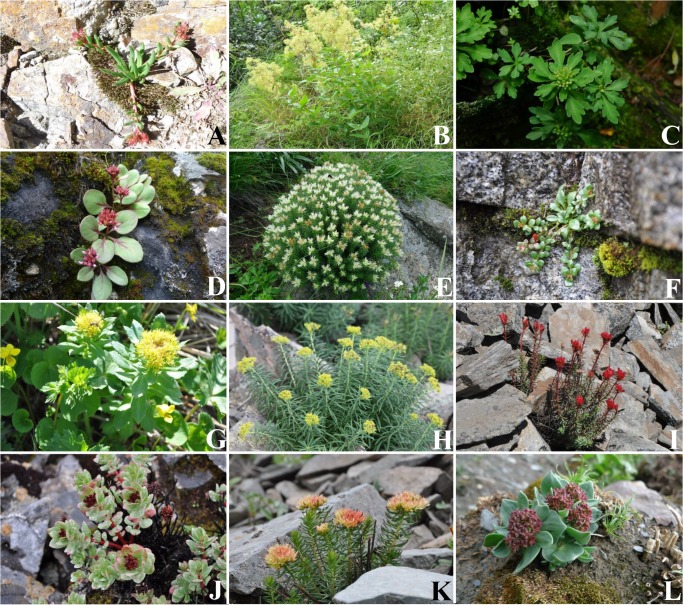
Representatives of species illustrating the morphological variation in Rhodiola. (A. *Rhodiola smithii*; B. *R*. *yunnanensis*; C. *R*. *chrysanthemifolia*; D. *R*. *prainii*; E. *R*. *dumulosa*; F. *R*. *hobsonii*; G. *R*. *rosea*; H. *R*. *kirilowii*; I. *R*. *fastigiata*; J. *R*. *crenulata*; K. *R*. *alsia*; L. *R*. *bupleuroides*).

**Fig 2:**
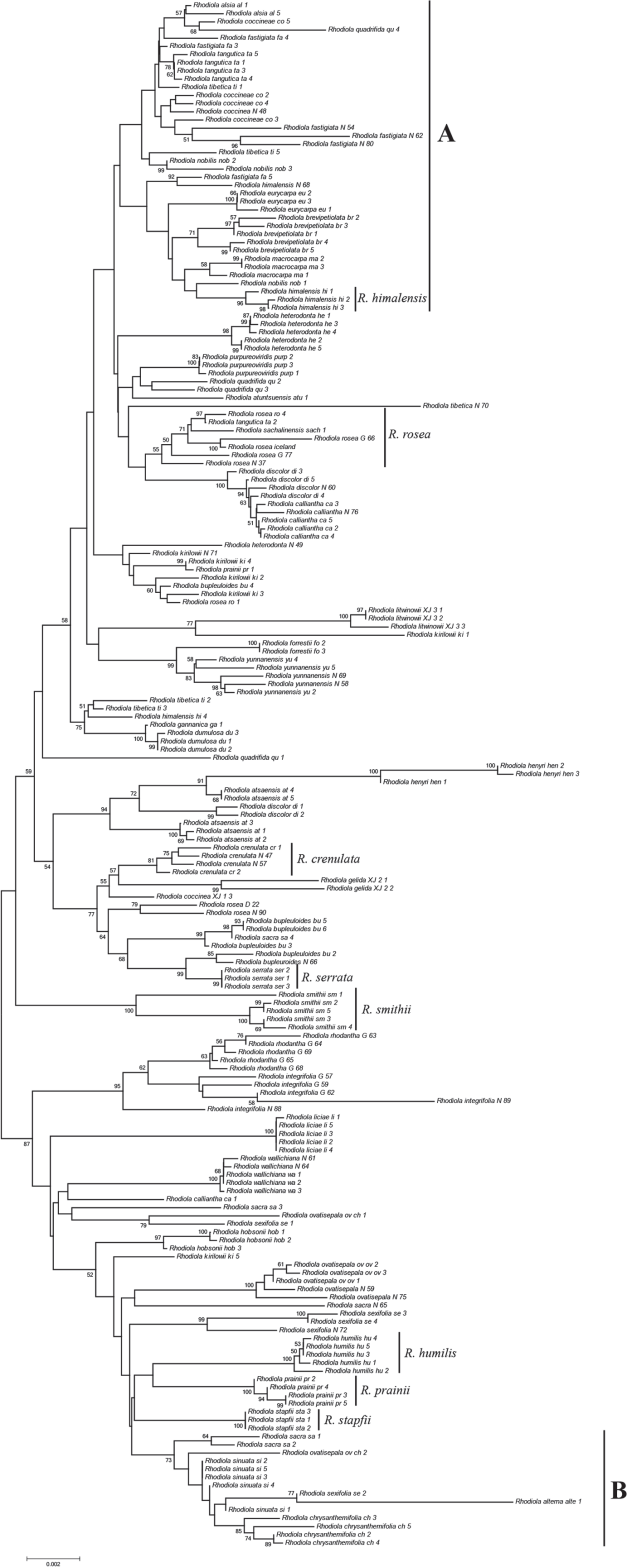
Neighbor joining tree using the Kimula 2-parameter distances based on all five barcoding markers for *Rhodiola* species. Numbers at nodes represent bootstrap values with 1000 replicates (only values > 50 were shown).

Extensive sampling and analysis of taxonomically well understood groups are needed to thoroughly validate and standardize markers and procedures for DNA barcoding [49]. A number of studies have tested the application of DNA barcoding in various plant groups from familial to generic levels [40–42,50–56]. Familial-level studies often included representatives of each genus [53,54,57]. In contrast, most generic level studies only examined a small fraction of species [40–42,51], or focused on a single species [55], with some exceptions [50]. Thus, a major objective of the present study was to test the candidate DNA barcodes at the generic level using an extensive taxon sampling scheme [58–62]. Both morphologically divergent (Fig. 1, A-F) and highly similar species (Fig. 1, I, K) were included in the data set. Not surprisingly, morphologically divergent species were easily identified by DNA barcodes, whereas species with highly similar morphological characters remained largely unresolved (Fig. 1). For example, *R*. *smithii* (Raym.-Hamet) S. H. Fu (Fig. 1A) is easily distinguished from other species of *Rhodiola* by its radical leaves with appendages on them, and accessions of the species were found to be monophyletic with high bootstrap support (Fig. 2). This is also the case for three other morphologically distinct species, *R*. *humilis* (Hook. f. & Thomson) S. H. Fu, *R*. *stapfii* (Raym.-Hamet) S. H. Fu and *R*. *prainii* (Raym.-Hamet) H. Ohba (Fig. 1, D). On the other hand, accessions of two morphologically similar species, *R*. *fastigiata* (Fig. 1, I) and *R*. *tibetica* (Hook. f. & Thomson) S. H. Fu were scattered in one of the *Rhodiola* clades (Fig. 2, Clade A). Furthermore, accessions of the species of *R*. sect. *Trifida* all mingled together on clade B (Fig. 2). Species of this section are highly similar morphologically and only show slight differences in leaf morphology [19].

### DNA barcoding in a recently diversified plant group

With the potential of DNA barcoding to facilitate species identification and discovery [49,63], its applicability in various plant groups has been evaluated recently [22,40,51]. Problems have been encountered in some cases due to complexities arising from the reproductive behavior and evolutionary history, such as interspecific hybridization, introgression, allopolyploidy, mixtures of sexual and asexual reproduction, and recent divergences [22,64]. Such biological factors may have blurred species boundaries in *Rhodiola*, which was dated to have diversified recently, with its crown group diverged ca. 6.32 Ma [20]. Furthermore, hybridization and introgression may have played important roles in its evolutionary history [20].

The complexities seen in *Rhodiola* are common in many plant groups, but their effects on DNA barcoding have not been rigorously assessed. The rate of successful species identification was lower than in other plant barcoding studies [40–42,50–56]. As shown in Table 3, at most 80.9% of the species were successfully recovered as monophyletic groups, even when using all of the markers (*rbcL + matK + trnH-psbA + trnL-F +* ITS), and at most 72% of the species were successfully identified using the TaxonDNA test (Table 5). We attribute the low discrimination power of the tested barcodes to two main factors. Firstly, the rapid recent species radiations of *Rhodiola* may have resulted in polytomies of the gene trees, preventing most markers from accumulating sufficient variation to distinguish different species reliably, even if they can be distinguished morphologically (Fig. 1). Secondly, incomplete lineage sorting (ILS) and reticulate evolution, which may occur alone or together, may have blurred species boundaries, impeding clear barcoding. ILS, caused by the retention of ancestral polymorphisms [65–67], is likely to lead to discordant and unpredictable associations between accessions of different species due to its stochastic nature. In contrast to ILS, reticulate evolution resulting from post-speciation hybridization and organelle capture among pairs of taxa may show systematic associations between species. Both stochastic and systematic associations between different accessions of species were observed (Fig. 2), indicating that both processes may have played a role in the evolutionary history of *Rhodiola*, leading to the complications to barcode species of this genus.

In contrast to animals, many plant species are likely to have paraphyletic or polyphyletic origins due to the higher frequency of reticulate evolution in plants, as facilitated by hybridization and polyploidization [68]. Under these circumstances, barcoding based solely on plastid markers may not reliably distinguish species [69]. However, nuclear DNA sequences, e.g., the internal transcribed spacer region (ITS), may improve the resolution among plant species due to its generally higher synonymous substitution rates [70] and less sensitivity to problems caused by hybridization [8]. Our results show that ITS can distinguish more species than the combination of four plastid markers (66.0% vs 61.7%, Table 3). Thus, our DNA barcoding analysis confirms that ITS, and probably other nuclear genes, are powerful tools for identifying plant species with complex evolutionary histories.

### Identification of medicinal plants using DNA barcodes

The present study included six species of *Rhodiola* reported with medicinal properties (*R*. *rosea*, *R*. *crenulata*, *R*. *sachalinensis*, *R*. *himalensis*, *R*. *serreta*, and *R*. *fastigiata*). As one of the most important traditional herbal remedies, *R*. *crenulata* has been used in treating long-term illnesses and weaknesses caused by infection in Tibet and other regions for more than 1000 years [13,17]. Four individuals of the species from four different geographic areas form a monophyletic group based on the five-marker barcodes (Fig. 2), showing the feasibility of using DNA barcoding to distinguish this species from other species of the genus or other adulterants. *Rhodiola rosea* has also been widely used in East Europe and Asia for stimulating the nervous system and decreasing depression [15]. In our analysis, five accessions of *R*. *rosea* from different localities formed a clade with the only accession of *R*. *sachalinensis*, and one accession of *R*. *tangutica*. *Rhodiola rosea* and *R*. *sachalinensis* have strong morphological similarity [10]. Two other species (*R*. *himalensis* and *R*. *serreta*) could also be correctly identified (Fig. 2). However, *R*. *fastigiata* can not be identified successfully using the five-marker barcode because of its high intraspecific variation, probably due to incomplete lineage sorting. In summary, five of the six plant species with medicinal properties are each identifiable using barcodes tested in the present study. Thus, the results indicate not only the potential utility of barcoding for these plants, but also the need to validate the barcodes and interpret the results carefully.

## Supporting Information

S1 Fig Distributions of intra- and inter-specific Kimura 2-parameter (K2P) distances for five candidate barcodes in *Rhodiola*. (PPTX)

S1 Table Localities, voucher information and GenBank accessions numbers for sequenced taxa. (DOC)
