# Supplementary figures and images for: DNA Barcoding of Rhodiola (Crassulaceae): A Case Study on a Group of Recently Diversified Medicinal Plants from the Qinghai-Tibetan Plateau
Source: PLoS One. 2015 Mar 16;10(3):e0119921. doi: 10.1371/journal.pone.0119921 (PMC4361186; doi:10.1371/journal.pone.0119921)

## Slide 1
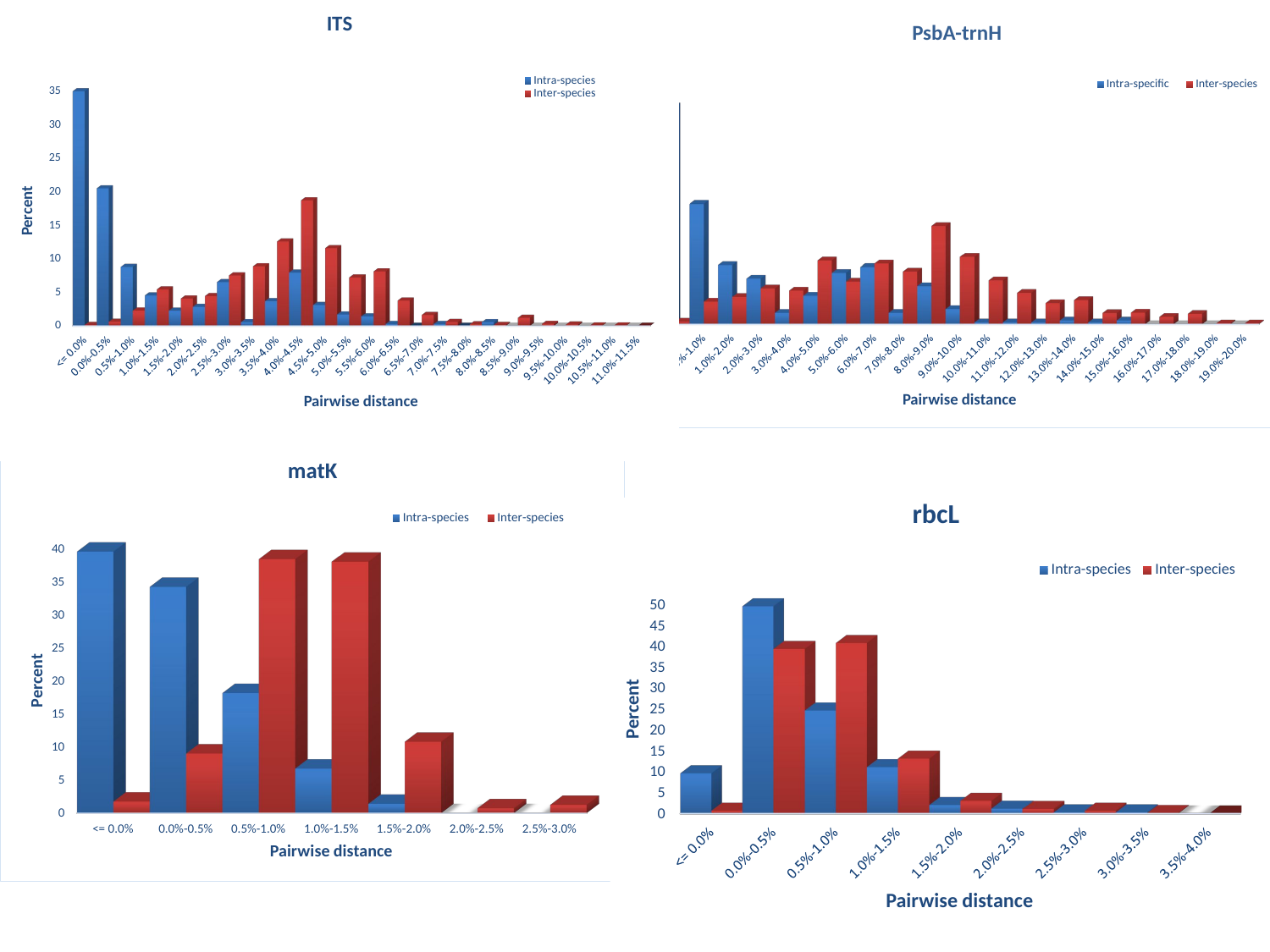

PsbA-trnH
#

## Slide 2
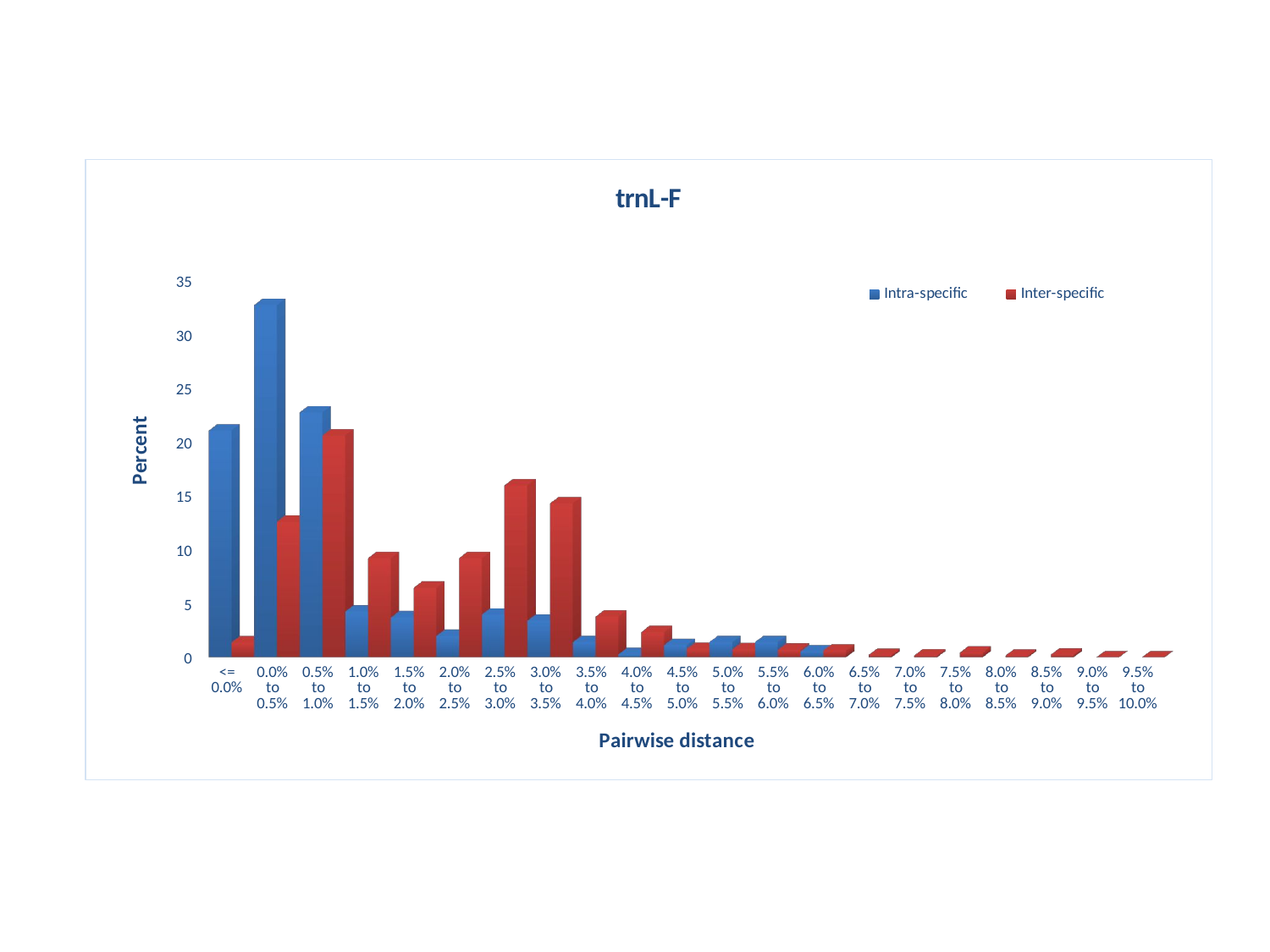

[unsupported chart]

Supplement: S1 Fig — (PPTX) [file pone.0119921.s001.pptx]
