# Supplementary material for: DNA Barcoding of Rhodiola (Crassulaceae): A Case Study on a Group of Recently Diversified Medicinal Plants from the Qinghai-Tibetan Plateau
Source: PLoS One. 2015 Mar 16;10(3):e0119921. doi: 10.1371/journal.pone.0119921 (PMC4361186; doi:10.1371/journal.pone.0119921)
Supplement: S1 Table — (DOC) [file pone.0119921.s002.doc]

Table S1. Localities, voucher information and GenBank accessions numbers for sequenced taxa.

| **Taxon** | **Location** | **Latitude (N)** | **Longitude (E)** | **Voucher** | **Collector** | **ITS** | ***trnL-F*** | ***psbA-trnH*** | ***matK*** | ***rbcL*** |
| --- | --- | --- | --- | --- | --- | --- | --- | --- | --- | --- |
| *Rhodiola alsia* (Fröd.) S. H. Fu | China. Qinghai: Yushu Xian | 32° 46' 30'' | 97° 03' 83'' | *J. Q. Zhang et al. 100829-04* (PEY) | Zhang Jian-Qiang et al. | KF113683 | KF113789 | KF113736 | KJ570428 | KJ570523 |
| *Rhodiola alsia* (Fröd.) S. H. Fu | China. Sichuan: Kangding Xian | 30° 04' 45'' | 101° 48' 23'' | *J. Q. Zhang et al. 110812-06* (PEY) | Zhang Jian-Qiang et al. | KP114694 | KP115100 | KP114889 | KP114802 | KP114997 |
| *Rhodiola alterna* S. H. Fu | China. Xizang: Baqing Xian | 31° 45' 12'' | 94° 28' 52'' | *G. Y. Rao 090803-01*(PEY) | Rao Guang-Yuan | KF113684 | KF113790 | KF113737 | KJ570429 | KJ570524 |
| *Rhodiola atsaensis* (Fröd.) H. Ohba | China. Sichuan: Kangding Xian | 30° 04' 09'' | 101° 48' 08'' | *J. Q. Zhang et al. 110812-04-01* (PEY) | Zhang Jian-Qiang et al. | KF113685 | KF113791 | KF113738 | KJ570430 | KJ570525 |
| *Rhodiola atsaensis* (Fröd.) H. Ohba | China. Sichuan: Kangding Xian | 30° 04' 11'' | 101° 48' 09'' | *J. Q. Zhang et al. 110812-04-05* (PEY) | Zhang Jian-Qiang et al. | KP114695 | KP115101 | KP114890 | KP114803 | KP114998 |
| *Rhodiola atsaensis* (Fröd.) H. Ohba | China. Sichuan: Kangding Xian | 30° 04' 08'' | 101° 48' 14'' | *J. Q. Zhang et al. 110812-04-03* (PEY) | Zhang Jian-Qiang et al. | KP114696 | KP115102 | KP114891 | KP114804 | KP114999 |
| *Rhodiola atsaensis* (Fröd.) H. Ohba | China. Sichuan: Kangding Xian | 30° 04' 06'' | 101° 48' 07'' | *J. Q. Zhang et al. 110812-04-02* (PEY) | Zhang Jian-Qiang et al. | KP114697 | KP115103 | KP114892 | KP114805 | KP115000 |
| *Rhodiola atsaensis* (Fröd.) H. Ohba | China. Sichuan: Kangding Xian | 30° 04' 15'' | 101° 48' 05'' | *J. Q. Zhang et al. 110812-04-04* (PEY) | Zhang Jian-Qiang et al. | KP114698 | KP115104 | KP114893 | KP114806 | KP115001 |
| *Rhodiola atuntsuensis* (Praeger) S. H. Fu | China. Xizang | 29° 42' 22'' | 98° 00' 25'' | *Tibet-MacArthur 2478* (US) | Wen Jun et al. | KJ569922 | KJ570288 | KJ570050 | KJ570431 | KJ570526 |
| *Rhodiola brevipetiolata* (Fröd.) S. H. Fu | China. Sichuan: Xiangcheng Xian | 29° 08' 21'' | 100° 03' 18'' | *J. Q. Zhang et al. 110809-06* (PEY) | Zhang Jian-Qiang et al. | KF113686 | KF113792 | KF113739 | KJ570432 | KJ570527 |
| *Rhodiola brevipetiolata* (Fröd.) S. H. Fu | China. Sichuan: Xiangcheng Xian | 29° 08' 13'' | 100° 03' 19'' | *J. Q. Zhang et al. 110809-06-04* (PEY) | Zhang Jian-Qiang et al. | KP114699 | KP115105 | KP114894 | KP114807 | KP115002 |
| *Rhodiola brevipetiolata* (Fröd.) S. H. Fu | China. Sichuan: Xiangcheng Xian | 29° 08' 19'' | 100° 03' 23'' | *J. Q. Zhang et al. 110809-06-09* (PEY) | Zhang Jian-Qiang et al. | KP114700 | KP115106 | KP114895 | - | KP115003 |
| *Rhodiola brevipetiolata* (Fröd.) S. H. Fu | China. Sichuan: Kangding Xian | 30° 04' 14'' | 101° 48' 14'' | *J. Q. Zhang et al. 110812-01-5c* (PEY) | Zhang Jian-Qiang et al. | KP114701 | KP115107 | KP114896 | KP114808 | KP115004 |
| *Rhodiola brevipetiolata* (Fröd.) S. H. Fu | China. Sichuan: Kangding Xian | 30° 04' 13'' | 101° 48' 12'' | *J. Q. Zhang et al. 110812-01-10* (PEY) | Zhang Jian-Qiang et al. | KP114702 | KP115108 | KP114897 | KP114809 | KP115005 |
| *Rhodiola bupleuroides* (Wall. ex Hook. f. & Thomson) S. H. Fu | China. Xizang: Gongbujiangda Xian | 29° 50' 18'' | 92° 19' 26'' | *G. Y. Rao 090728-02* (PEY) | Rao Guang-Yuan | KP114703 | KP115109 | KP114898 | - | KP115006 |
| *Rhodiola bupleuroides* (Wall. ex Hook. f. & Thomson) S. H. Fu | China. Xizang: Jiangzi Xian | 28° 53' 38'' | 90° 10' 18'' | *G. Y. Rao 090729-03* (PEY) | Rao Guang-Yuan | KF113687 | KF113793 | KF113740 | KJ570433 | KJ570528 |
| *Rhodiola bupleuroides* (Wall. ex Hook. f. & Thomson) S. H. Fu | China. Xizang: Baqing Xian | 31° 45' 12'' | 94° 28' 52'' | *G. Y. Rao 090803-02* (PEY) | Rao Guang-Yuan | KP114704 | KP115110 | KP114899 | KP114810 | KP115007 |
| *Rhodiola bupleuroides* (Wall. ex Hook. f. & Thomson) S. H. Fu | China. Xizang: Lazi Xian | 28° 56' 20'' | 87° 21' 20'' | *G. Y. Rao et al. 100813-03* (PEY) | Rao Guang-Yuan et al. | KP114705 | KP115111 | KP114900 | - | KP115008 |
| *Rhodiola bupleuroides* (Wall. ex Hook. f. & Thomson) S. H. Fu | China. Xizang: Lazi Xian | 28° 56' 31'' | 87° 23' 19'' | *G. Y. Rao et al. 100813-04* (PEY) | Rao Guang-Yuan et al. | KP114706 | KP115112 | KP114901 | - | KP115009 |
| *Rhodiola bupleuroides* (Wall. ex Hook. f. & Thomson) S. H. Fu | China. Xizang | 30° 07' 12'' | 92° 09' 26'' | *Tibet-MacArthur 110* (US) | Wen Jun et al. | KJ569923 | KJ570289 | KJ570051 | KJ570434 | KJ570529 |
| *Rhodiola calliantha* (H. Ohba) H. Ohba | China. Xizang: Nielamu Xian | 28° 06' 32'' | 85° 57' 27'' | *G. Y. Rao et al. 100814-01-00* (PEY) | Rao Guang-Yuan et al. | KP114707 | KP115113 | KP114902 | KP114811 | KP115010 |
| *Rhodiola calliantha* (H. Ohba) H. Ohba | China. Xizang: Nielamu Xian | 28° 06' 39'' | 85° 57' 30'' | *G. Y. Rao et al. 100814-01-06*(PEY) | Rao Guang-Yuan et al. | KP114708 | KP115114 | KP114903 | - | KP115011 |
| *Rhodiola calliantha* (H. Ohba) H. Ohba | China. Xizang: Nielamu Xian | 28° 06' 42'' | 85° 57' 31'' | *G. Y. Rao et al. 100814-01-09*(PEY) | Rao Guang-Yuan et al. | KP114709 | KP115115 | KP114904 | KP114812 | KP115012 |
| *Rhodiola calliantha* (H. Ohba) H. Ohba | China. Xizang: Nielamu Xian | 28° 06' 37'' | 85° 57' 33'' | *G. Y. Rao et al. 100814-01-03*(PEY) | Rao Guang-Yuan et al. | KP114710 | KP115116 | KP114905 | KP114813 | KP115013 |
| *Rhodiola calliantha* (H. Ohba) H. Ohba | China. Xizang: Nielamu Xian | 28° 06' 25'' | 85° 57' 39'' | *G. Y. Rao et al. 100814-01-01*(PEY) | Rao Guang-Yuan et al. | KF113688 | KF113794 | KF113741 | KJ570435 | KJ570530 |
| *Rhodiola calliantha* (H. Ohba) H. Ohba | China. Xizang | 27° 50' 37'' | 85° 48' 41'' | *Tibet-MacArthur 750* (US) | Wen Jun et al. | KJ569924 | KJ570290 | KJ570052 | KJ570436 | KJ570531 |
| *Rhodiola chrysanthemifolia* (H. Lév.) S. H. Fu | China. Yunnan: Baima Mt. | 28° 21' 25'' | 99° 02' 88'' | *J. Q. Zhang et al. 110803-06-03* (PEY) | Zhang Jian-Qiang et al. | KP114711 | KP115117 | KP114906 | - | KP115014 |
| *Rhodiola chrysanthemifolia* (H. Lév.) S. H. Fu | China. Yunnan: Baima Mt. | 28° 21' 23'' | 99° 02' 81'' | *J. Q. Zhang et al. 110803-06-06* (PEY) | Zhang Jian-Qiang et al. | KF113689 | KF113795 | KF113742 | KJ570437 | KJ570532 |
| *Rhodiola chrysanthemifolia* (H. Lév.) S. H. Fu | China. Yunnan: Baima Mt. | 28° 21' 24'' | 99° 02' 68'' | *J. Q. Zhang et al. 110803-06-01* (PEY) | Zhang Jian-Qiang et al. | KP114712 | KP115118 | KP114907 | - | KP115015 |
| *Rhodiola chrysanthemifolia* (H. Lév.) S. H. Fu | China. Yunnan: Baima Mt. | 28° 21' 28'' | 99° 02' 92'' | *J. Q. Zhang et al. 110803-06-08* (PEY) | Zhang Jian-Qiang et al. | KP114713 | KP115119 | KP114908 | - | KP115016 |
| *Rhodiola coccinea* (Royle) Boriss | China. Xinjiang: Zhaosu Xian | 43° 23' 48'' | 81° 02' 14'' | *G. Y. Rao et al. 120829-01* (PEY) | Rao Guang-Yuan et al. | KP114714 | KP115120 | KP114909 | KP114814 | KP115017 |
| *Rhodiola coccinea* (Royle) Boriss | China. Xizang: Lazi Xian | 28° 56' 27'' | 87° 23' 31'' | *G. Y. Rao 100813-02* (PEY) | Rao Guang-Yuan | KF113690 | KF113796 | KF113743 | KJ570438 | KJ570533 |
| *Rhodiola coccinea* (Royle) Boriss | China. Xizang: Lazi Xian | 28° 56' 21'' | 87° 23' 44'' | *G. Y. Rao 100813-02-02* (PEY) | Rao Guang-Yuan | KP114715 | KP115121 | KP114910 | KP114815 | KP115018 |
| *Rhodiola coccinea* (Royle) Boriss | China. Xizang: Lazi Xian | 28° 56' 37'' | 87° 23' 32'' | *G. Y. Rao 100813-02-04* (PEY) | Rao Guang-Yuan | KP114716 | KP115122 | KP114911 | KP114816 | KP115019 |
| *Rhodiola coccinea* (Royle) Boriss | China. Sichuan: Kangding Xian | 30° 04' 11'' | 101° 48' 12'' | *J. Q. Zhang et al. 110812-03-08* (PEY) | Zhang Jian-Qiang et al. | KP114717 | KP115123 | KP114912 | KP114817 | KP115020 |
| *Rhodiola coccinea* (Royle) Boriss | China. Xizang | 30° 31' 11'' | 91° 06' 25'' | *Tibet-MacArthur 3417* (US) | Wen Jun et al. | KJ569925 | KJ570291 | KJ570053 | KJ570439 | KJ570534 |
| *Rhodiola crenulata* (Hook. f. & Thomson) H. Ohba | China. Sichuan: Dege Xian | 31° 56' 02'' | 98° 56' 02'' | *G. Y. Rao 090722-01* (PEY) | Rao Guang-Yuan | KP114718 | KP115124 | KP114913 | - | KP115021 |
| *Rhodiola crenulata* (Hook. f. & Thomson) H. Ohba | China. Xizang: Lazi Xian | 28° 57' 28'' | 87° 23' 37'' | *G. Y. Rao et al. 100813-01* (PEY) | Rao Guang-Yuan et al. | KF113691 | KF113797 | KF113744 | KJ570440 | KJ570535 |
| *Rhodiola crenulata* (Hook. f. & Thomson) H. Ohba | China. Xizang | 29° 43' 19'' | 98° 00' 25'' | *Tibet-MacArthur 2474* (US) | Wen Jun et al. | KJ569926 | KJ570292 | KJ570054 | KJ570441 | KJ570536 |
| *Rhodiola crenulata* (Hook. f. & Thomson) H. Ohba | China. Xizang | 29° 50' 14'' | 92° 19' 32'' | *Tibet-MacArthur 3256* (US) | Wen Jun et al. | KJ569927 | KJ570293 | KJ570055 | KJ570442 | KJ570537 |
| *Rhodiola discolor* (Franch.) S. H. Fu | China. Xizang: Jiangda Xian | 31° 35' 31'' | 98° 34' 31'' | *G. Y. Rao 090723-01* (PEY) | Rao Guang-Yuan | KF113692 | KF113798 | KF113745 | KJ570443 | KJ570538 |
| *Rhodiola discolor* (Franch.) S. H. Fu | China. Xizang: Jiangda Xian | 31° 35' 31'' | 98° 34' 31'' | *G. Y. Rao 090723-02* (PEY) | Rao Guang-Yuan | KP114719 | KP115125 | KP114914 | KP114818 | KP115022 |
| *Rhodiola discolor* (Franch.) S. H. Fu | China. Xizang: Nielamu Xian | 28° 07' 21'' | 85° 58' 02'' | *J. Q. Zhang 100815-03* (PEY) | Zhang Jian-Qiang et al. | KP114720 | KP115126 | KP114915 | KP114819 | KP115023 |
| *Rhodiola discolor* (Franch.) S. H. Fu | China. Xizang: Jilong Xian | 28° 29' 27'' | 85° 14' 19'' | *J. Q. Zhang 100818-03* (PEY) | Zhang Jian-Qiang et al. | KP114721 | KP115127 | KP114916 | KP114820 | KP115024 |
| *Rhodiola discolor* (Franch.) S. H. Fu | China. Xizang: Jilong Xian | 28° 30' 58'' | 85° 13' 10'' | *J. Q. Zhang 100818-04* (PEY) | Zhang Jian-Qiang et al. | KP114722 | KP115128 | KP114917 | KP114821 | KP115025 |
| *Rhodiola discolor* (Franch.) S. H. Fu | China. Xizang | 28° 06' 01'' | 85° 59' 25'' | *Tibet-MacArthur 776* (US) | Wen Jun et al. | KJ569928 | KJ570294 | KJ570056 | KJ570444 | KJ570539 |
| *Rhodiola dumulosa* (Franch.) S. H. Fu | China. Beijing: Dongling Mt. | 39° 59' 82'' | 115° 25' 37'' | *J. Q. Zhang 100717-05* (PEY) | Zhang Jian-Qiang et al. | KF113693 | KF113799 | KF113746 | KJ570445 | KJ570540 |
| *Rhodiola dumulosa* (Franch.) S. H. Fu | China. Beijing: Dongling Mt. | 39° 59' 87'' | 115° 25' 34'' | *J. Q. Zhang 100717-06* (PEY) | Zhang Jian-Qiang et al. | KP114723 | KP115129 | KP114918 | KP114822 | KP115026 |
| *Rhodiola dumulosa* (Franch.) S. H. Fu | China. Beijing: Dongling Mt. | 39° 59' 74'' | 115° 25' 87'' | *J. Q. Zhang 100717-07* (PEY) | Zhang Jian-Qiang et al. | KP114724 | KP115130 | KP114919 | KP114823 | KP115027 |
| *Rhodiola eurycarpa* (Franch.) S. H. Fu | China. Gansu: Lianhua Mt. | 34° 56' 42'' | 103° 45' 58'' | *J. Q. Zhang 120705-03-10* (PEY) | Zhang Jian-Qiang et al. | KF113694 | KF113800 | KF113747 | KJ570446 | KJ570541 |
| *Rhodiola eurycarpa* (Franch.) S. H. Fu | China. Gansu: Lianhua Mt. | 34° 56' 47'' | 103° 45' 59'' | *J. Q. Zhang 120705-03-03* (PEY) | Zhang Jian-Qiang et al. | KP114725 | KP115131 | KP114920 | KP114824 | KP115028 |
| *Rhodiola eurycarpa* (Franch.) S. H. Fu | China. Gansu: Lianhua Mt. | 34° 56' 43'' | 103° 46' 10'' | *J. Q. Zhang 120705-03-12* (PEY) | Zhang Jian-Qiang et al. | KP114726 | KP115132 | KP114921 | KP114825 | KP115029 |
| *Rhodiola fastigiata* (Hook. f. & Thomson) S. H. Fu | China. Xizang: Linzhi Xian | 29° 37' 58'' | 94° 37' 30'' | *G. Y. Rao 090727-01*(PEY) | Rao Guang-Yuan | KF113695 | KF113801 | KF113748 | KJ570447 | KJ570542 |
| *Rhodiola fastigiata* (Hook. f. & Thomson) S. H. Fu | China. Xizang: Mala Mt. | 28° 54' 14'' | 85° 22' 64'' | *G. Y. Rao et al. 100819-01* (PEY) | Rao Guang-Yuan et al. | KP114727 | KP115133 | KP114922 | KP114826 | KP115030 |
| *Rhodiola fastigiata* (Hook. f. & Thomson) S. H. Fu | [China. Yunnan: Shangri-La Xian](app:ds:Shangri-La) | 28° 07' 09'' | 99° 54' 04'' | *J. Q. Zhang 110731-01* (PEY) | Zhang Jian-Qiang et al. | KP114728 | KP115134 | KP114923 | - | KP115031 |
| *Rhodiola fastigiata* (Hook. f. & Thomson) S. H. Fu | China. Xizang | 29° 42' 58'' | 95° 42' 01'' | *Tibet-MacArthur 2697* (US) | Wen Jun et al. | KJ569930 | KJ570296 | KJ570058 | KJ570449 | KJ570544 |
| *Rhodiola fastigiata* (Hook. f. & Thomson) S. H. Fu | China. Xizang | 29° 43' 05'' | 94° 42' 51'' | *Tibet-MacArthur 3021* (US) | Wen Jun et al. | KJ569931 | KJ570297 | KJ570059 | KJ570450 | KJ570545 |
| *Rhodiola fastigiata* (Hook. f. & Thomson) S. H. Fu | China. Xizang | 29° 42' 56'' | 94° 36' 01'' | *Tibet-MacArthur 695* (US) | Wen Jun et al. | KJ569932 | KJ570298 | KJ570060 | KJ570451 | KJ570546 |
| *Rhodiola forrestii* (Raym.-Hamet) S. H. Fu | [China. Yunnan: Shangri-La Xian](app:ds:Shangri-La) | 27° 53' 58'' | 99° 33' 46'' | *J. Q. Zhang et al. 110730-02-01*(PEY) | Zhang Jian-Qiang et al. | KF113696 | KF113802 | KF113749 | KJ570452 | KJ570547 |
| *Rhodiola forrestii* (Raym.-Hamet) S. H. Fu | [China. Yunnan: Shangri-La Xian](app:ds:Shangri-La) | 27° 53' 40'' | 99° 33' 48'' | *J. Q. Zhang et al. 110730-02-02*(PEY) | Zhang Jian-Qiang et al. | KP114729 | KP115135 | KP114924 | KP114827 | KP115032 |
| *Rhodiola gelida* Schrenk | China. Xinjiang | 43° 23' 21'' | 81° 02' 10'' | *G. Y. Rao et al. 120829-02-05* (PEY) | Rao Guang-Yuan et al. | KP114730 | KP115136 | KP114925 | KP114828 | - |
| *Rhodiola gannanica* K. T. Fu | China. Gannan Prefecture | 34° 56' 71'' | 102° 55' 87'' | *G. Y. Rao et al. 100731-01* (PEY) | Rao Guang-Yuan et al. | KF113697 | KF113803 | KF113750 | KJ570453 | KJ570548 |
| *Rhodiola gelida* Schrenk | China. Xinjiang | 43° 23' 21'' | 81° 02' 10'' | *G. Y. Rao et al. 120829-02-04* (PEY) | Rao Guang-Yuan et al. | KJ569933 | KJ570299 | KJ570061 | KJ570454 | KJ570549 |
| *Rhodiola henryi* (Diels) S. H. Fu | China. Shannxi: Taibai Xian | 34° 01' 50'' | 107° 51' 46'' | *J. Q. Zhang et al. 120820-04-01* (PEY) | Zhang Jian-Qiang et al. | KF113698 | KF113804 | KF113751 | KJ570455 | KJ570550 |
| *Rhodiola henryi* (Diels) S. H. Fu | China. Shannxi: Taibai Xian | 34° 01' 58'' | 107° 52' 42'' | *J. Q. Zhang et al. 120820-04-02* (PEY) | Zhang Jian-Qiang et al. | KP114731 | KP115137 | KP114926 | - | - |
| *Rhodiola henryi* (Diels) S. H. Fu | China. Shannxi: Taibai Xian | 34° 01' 49'' | 107° 52' 47'' | *J. Q. Zhang et al. 120820-04-03* (PEY) | Zhang Jian-Qiang et al. | KP114732 | KP115138 | KP114927 | - | - |
| *Rhodiola heterodonta* (Hook. f. & Thomson) Boriss. | China. Xizang: Nanmulin Xian | 30° 04' 47'' | 89° 06' 25'' | *G. Y. Rao et al. 100811-02* (PEY) | Rao Guang-Yuan et al. | KF113699 | KF113805 | KF113752 | KJ570456 | KJ570551 |
| *Rhodiola heterodonta* (Hook. f. & Thomson) Boriss. | China. Xizang: Nielamu Xian | 28° 04' 17'' | 85° 56' 21'' | *G. Y. Rao et al. 100816-01-00* (PEY) | Rao Guang-Yuan et al. | KP114733 | KP115139 | KP114928 | KP114829 | KP115033 |
| *Rhodiola heterodonta* (Hook. f. & Thomson) Boriss. | China. Xizang: Nanmulin Xian | 30° 04' 47'' | 89° 06' 25'' | *G. Y. Rao et al. 100811-02-03* (PEY) | Rao Guang-Yuan et al. | KP114734 | KP115140 | KP114929 | KP114830 | KP115034 |
| *Rhodiola heterodonta* (Hook. f. & Thomson) Boriss. | China. Xizang: Nanmulin Xian | 30° 04' 47'' | 89° 06' 25'' | *G. Y. Rao et al. 100811-02-05* (PEY) | Rao Guang-Yuan et al. | KP114735 | KP115141 | KP114930 | KP114831 | KP115035 |
| *Rhodiola heterodonta* (Hook. f. & Thomson) Boriss. | China. Xizang: Nielamu Xian | 28° 04' 17'' | 85° 56' 21'' | *G. Y. Rao et al. 100816-01-04* (PEY) | Rao Guang-Yuan et al. | KP114736 | KP115142 | KP114931 | KP114832 | KP115036 |
| *Rhodiola heterodonta* (Hook. f. & Thomson) Boriss. | China. Xizang | 29° 42' 26'' | 94° 42' 35'' | *Tibet-MacArthur 3053* (US) | Wen Jun et al. | KJ569934 | KJ570300 | KJ570062 | KJ570457 | KJ570552 |
| *Rhodiola himalensis* (D. Don) S. H. Fu | China. Xizang: Nielamu Xian | 28° 08' 44'' | 85° 58' 31'' | *G. Y. Rao et al. 100815-01* (PEY) | Rao Guang-Yuan et al. | KF113700 | KF113806 | KF113753 | KJ570458 | KJ570553 |
| *Rhodiola himalensis* (D. Don) S. H. Fu | China. Xizang: Nielamu Xian | 28° 07' 45'' | 85° 58' 25'' | *G. Y. Rao et al. 100815-01-03* (PEY) | Rao Guang-Yuan et al. | KP114737 | KP115143 | KP114932 | KP114833 | KP115037 |
| *Rhodiola himalensis* (D. Don) S. H. Fu | China. Xizang: Nielamu Xian | 28° 08' 42'' | 85° 58' 39'' | *G. Y. Rao et al. 100815-01-05* (PEY) | Rao Guang-Yuan et al. | KP114738 | KP115144 | KP114933 | KP114834 | KP115038 |
| *Rhodiola himalensis* (D. Don) S. H. Fu | China. Xizang: Nielamu Xian | 28° 08' 59'' | 85° 58' 42'' | *G. Y. Rao et al. 100815-01-02* (PEY) | Rao Guang-Yuan et al. | KP114739 | KP115145 | KP114934 | KP114835 | KP115039 |
| *Rhodiola himalensis* (D. Don) S. H. Fu | China. Yunnan | 28° 09' 10'' | 99° 54' 53'' | *Tibet-MacArthur 1149* (US) | Wen Jun et al. | KJ569935 | KJ570301 | KJ570063 | KJ570459 | KJ570554 |
| *Rhodiola hobsonii* (Prain ex ­Hamet) S. H. Fu | China. Xizang: Linzhi Xian | 29° 33' 80'' | 94° 34' 57'' | *J. Q. Zhang et al. 120717-05* (PEY) | Zhang Jian-Qiang et al. | KF113701 | KF113807 | KF113754 | KJ570460 | KJ570555 |
| *Rhodiola hobsonii* (Prain ex ­Hamet) S. H. Fu | China. Xizang: Linzhi Xian | 29° 33' 72'' | 94° 34' 59'' | *J. Q. Zhang et al. 120717-05-03* (PEY) | Zhang Jian-Qiang et al. | KP114740 | KP115146 | KP114935 | KP114836 | KP115040 |
| *Rhodiola hobsonii* (Prain ex ­Hamet) S. H. Fu | China. Xizang: Linzhi Xian | 29° 33' 85'' | 94° 34' 39'' | *J. Q. Zhang et al. 120717-05-02* (PEY) | Zhang Jian-Qiang et al. | KP114741 | KP115147 | KP114936 | KP114837 | KP115041 |
| *Rhodiola humilis* (HK. f. et Thoms.) S. H. Fu | China. Xizang: Mangkang Xian | 29° 41' 58'' | 98° 35' 31'' | *J. Q. Zhang et al. 110804-03* (PEY) | Zhang Jian-Qiang et al. | KF113702 | KF113808 | KF113755 | KJ570461 | KJ570556 |
| *Rhodiola humilis* (HK. f. et Thoms.) S. H. Fu | China. Xizang: Mangkang Xian | 29° 41' 52'' | 98° 35' 32'' | *J. Q. Zhang et al. 110804-03-03* (PEY) | Zhang Jian-Qiang et al. | KP114742 | KP115148 | KP114937 | KP114838 | KP115042 |
| *Rhodiola humilis* (HK. f. et Thoms.) S. H. Fu | China. Xizang: Mangkang Xian | 29° 41' 47'' | 98° 35' 36'' | *J. Q. Zhang et al. 110804-03-09* (PEY) | Zhang Jian-Qiang et al. | KP114743 | KP115149 | KP114938 | KP114839 | KP115043 |
| *Rhodiola humilis* (HK. f. et Thoms.) S. H. Fu | China. Xizang: Mangkang Xian | 29° 41' 42'' | 98° 35' 25'' | *J. Q. Zhang et al. 110804-03-02* (PEY) | Zhang Jian-Qiang et al. | KP114744 | KP115150 | KP114939 | KP114840 | KP115044 |
| *Rhodiola humilis* (HK. f. et Thoms.) S. H. Fu | China. Xizang: Mangkang Xian | 29° 41' 58'' | 98° 35' 41'' | *J. Q. Zhang et al. 110804-03-04* (PEY) | Zhang Jian-Qiang et al. | KP114745 | KP115151 | KP114940 | KP114841 | KP115045 |
| *Rhodiola integrifolia* Raf. | USA. Alaska | 65° 02' 36'' | -144° 52' 06'' | *J. E. Cantlon & W. T. Gillis 57-1657* (US) | Cantlon J. E. and Gillis W. T. | KJ569936 | KJ570302 | KJ570064 | KJ570462 | KJ570557 |
| *Rhodiola integrifolia* Raf. | USA. Alaska | 66° 27' 31'' | -145° 50' 19'' | *H. T. Shacklette 6294* (US) | Hansford T. Shacklette | - | KJ570303 | - | KJ570463 | - |
| *Rhodiola integrifolia* Raf. | USA. Alaska | 65° 24' 36'' | -145° 59' 06'' | *H. Guest 5182*(UVIC) | Heidi Guest | KF113703 | KF113809 | KF113756 | KJ570464 | KJ570558 |
| *Rhodiola integrifolia* Raf. | Canada. Yukon Territory | 64° 47' 58'' | -138° 03' 49'' | *H. Guest 5046* (UVIC) | Heidi Guest | KF113704 | KF113810 | KF113757 | KJ570465 | KJ570559 |
| *Rhodiola integrifolia* Raf. | USA. Wyoming | 41° 21' 35'' | -106° 18' 36'' | *H. Guest 6025* (UVIC) | Heidi Guest | KJ569940 | KJ570306 | KJ570068 | KJ570469 | KJ570563 |
| *Rhodiola kirilowii* (Regel) Maxim. | China. Sichuan: Luhuo Xian | 31° 43' 50'' | 100° 44' 09'' | *G. Y. Rao 090721-01* (PEY) | Rao Guang-Yuan | KP114746 | KP115152 | KP114941 | KP114842 | KP115046 |
| *Rhodiola kirilowii* (Regel) Maxim. | China. Sichuan: Luhuo Xian | 31° 42' 54'' | 100° 44' 01'' | *G. Y. Rao 090721-02* (PEY) | Rao Guang-Yuan | KP114747 | KP115153 | KP114942 | - | KP115047 |
| *Rhodiola kirilowii* (Regel) Maxim. | China. Xizang: Leiwuqi Xian | 31° 31' 07'' | 96° 21' 27'' | *G. Y. Rao 090806-01* (PEY) | Rao Guang-Yuan | KP114748 | KP115154 | KP114943 | - | KP115048 |
| *Rhodiola kirilowii* (Regel) Maxim. | China. Beijing: Dongling Mt. | 39° 59' 34'' | 115° 25' 28'' | *J. Q. Zhang 100717-01-RZ* (PEY) | Zhang Jian-Qiang et al. | KF113705 | KF113811 | KF113758 | KJ570470 | KJ570564 |
| *Rhodiola kirilowii* (Regel) Maxim. | China. Qinghai: Yushu Xian | 32° 51' 19'' | 97° 08' 73'' | *J. Q. Zhang 100829-01* (PEY) | Zhang Jian-Qiang et al. | KP114749 | KP115155 | KP114944 | KP114843 | KP115049 |
| *Rhodiola kirilowii* (Regel) Maxim. | China. Xizang | 29° 59' 51'' | 94° 11' 15'' | *Tibet-MacArthur 3223* (US) | Wen Jun et al. | KJ569941 | KJ570307 | KJ570069 | KJ570471 | KJ570565 |
| *Rhodiola liciae* (Raym.-Hamet) S. H. Fu | China. Yunnan: Xishan Mt. | 24° 56' 90'' | 102° 38' 37'' | *J. Q. Zhang et al. 110723-01* (PEY) | Zhang Jian-Qiang et al. | KF113706 | KF113812 | KF113759 | KJ570472 | KJ570566 |
| *Rhodiola liciae* (Raym.-Hamet) S. H. Fu | China. Yunnan: Xishan Mt. | 24° 55' 43'' | 102° 38' 32'' | *J. Q. Zhang et al. 110723-01-17* (PEY) | Zhang Jian-Qiang et al. | KP114750 | KP115156 | KP114945 | KP114844 | KP115050 |
| *Rhodiola liciae* (Raym.-Hamet) S. H. Fu | China. Yunnan: Xishan Mt. | 24° 54' 26'' | 102° 38' 65'' | *J. Q. Zhang et al. 110723-01-18* (PEY) | Zhang Jian-Qiang et al. | KP114751 | KP115157 | KP114946 | KP114845 | KP115051 |
| *Rhodiola liciae* (Raym.-Hamet) S. H. Fu | China. Yunnan: Xishan Mt. | 24° 56' 92'' | 102° 38' 24'' | *J. Q. Zhang et al. 110723-01-22* (PEY) | Zhang Jian-Qiang et al. | KP114752 | KP115158 | KP114947 | KP114846 | KP115052 |
| *Rhodiola liciae* (Raym.-Hamet) S. H. Fu | China. Yunnan: Xishan Mt. | 24° 56' 07'' | 102° 38' 58'' | *J. Q. Zhang et al. 110723-01-07* (PEY) | Zhang Jian-Qiang et al. | KP114753 | KP115159 | KP114948 | KP114847 | KP115053 |
| *Rhodiola litwinowii* Boriss. | China. Xinjiang | 42° 55' 78'' | 86° 09' 77'' | *G. Y. Rao et al. 120831-01-08* (PEY) | Rao Guang-Yuan et al. | KJ569942 | KJ570308 | KJ570070 | KJ570473 | - |
| *Rhodiola litwinowii* Boriss. | China. Xinjiang | 42° 55' 41'' | 86° 09' 72'' | *G. Y. Rao et al. 120831-01-04* (PEY) | Rao Guang-Yuan et al. | KP114754 | KP115160 | KP114949 | KP114848 | - |
| *Rhodiola litwinowii* Boriss. | China. Xinjiang | 42° 54' 59'' | 86° 09' 54'' | *G. Y. Rao et al. 120831-01-01* (PEY) | Rao Guang-Yuan et al. | KP114755 | KP115161 | KP114950 | KP114849 | - |
| *Rhodiola macrocarpa* (Praeger) S. H. Fu | China. Sichuan: Maerkang Xian | 31° 51' 80'' | 101° 20' 29'' | *J. Q. Zhang et al. 120709-07-03* (PEY) | Zhang Jian-Qiang et al. | KF113707 | KF113813 | KF113760 | KJ570474 | KJ570567 |
| *Rhodiola macrocarpa* (Praeger) S. H. Fu | China. Sichuan: Maerkang Xian | 31° 51' 56'' | 101° 20' 21'' | *J. Q. Zhang et al. 120709-07-01* (PEY) | Zhang Jian-Qiang et al. | KP114756 | KP115162 | KP114951 | KP114850 | KP115054 |
| *Rhodiola macrocarpa* (Praeger) S. H. Fu | China. Sichuan: Maerkang Xian | 31° 52' 08'' | 101° 20' 18'' | *J. Q. Zhang et al. 120709-07-08* (PEY) | Zhang Jian-Qiang et al. | KP114757 | KP115163 | KP114952 | KP114851 | KP115055 |
| *Rhodiola nobilis* (Franch.) S. H. Fu | China. Xizang: Qusong Xian | 29° 01' 39'' | 92° 21' 53'' | *J. Q. Zhang 120722-09-01* (PEY) | Zhang Jian-Qiang et al. | KF113708 | KF113814 | KF113761 | KJ570475 | KJ570568 |
| *Rhodiola nobilis* (Franch.) S. H. Fu | China. Xizang: Qusong Xian | 29° 01' 37'' | 92° 21' 58'' | *J. Q. Zhang 120722-09-06* (PEY) | Zhang Jian-Qiang et al. | KP114758 | KP115164 | KP114953 | KP114852 | KP115056 |
| *Rhodiola nobilis* (Franch.) S. H. Fu | China. Xizang: Qusong Xian | 29° 01' 18'' | 92° 21' 41'' | *J. Q. Zhang 120722-09-02* (PEY) | Zhang Jian-Qiang et al. | KP114759 | KP115165 | KP114954 | KP114853 | KP115057 |
| *Rhodiola ovatisepala* var. *ovatisepala* (Raym.-Hamet) S. H. Fu | China. Xizang | 28° 45' 23'' | 85° 32' 59'' | *Tibet-MacArthur 798* (US) | Wen Jun et al. | - | KJ570309 | KJ570071 | KJ570476 | - |
| *Rhodiola ovatisepala* var. *ovatisepala* (Raym.-Hamet) S. H. Fu | China. Xizang | 28° 01' 02'' | 85° 48' 26'' | *Tibet-MacArthur 902* (US) | Wen Jun et al. | - | KJ570310 | KJ570072 | KJ570477 | KJ570569 |
| *Rhodiola ovatisepala var.chingii* S. H. Fu | [China. Yunnan: Shangri-La Xian](app:ds:Shangri-La) | 28° 06' 80'' | 99° 48' 02'' | *J. Q. Zhang et al. 110731-05* (PEY) | Zhang Jian-Qiang et al. | KF113710 | KF113816 | KF113763 | KJ570478 | KJ570570 |
| *Rhodiola ovatisepala var.chingii* S. H. Fu | China. Sichuan: Xiangcheng Xian | 29° 07' 43'' | 100° 04' 32'' | *J. Q. Zhang et al. 110809-03* (PEY) | Zhang Jian-Qiang et al. | KP114760 | KP115166 | KP114955 | - | KP115058 |
| *Rhodiola ovatisepala* var. *ovatisepala* (Raym.-Hamet) S. H. Fu | China. Xizang: Nielamu Xian | 28° 05' 31'' | 85° 57' 32'' | *G. Y. Rao et al. 100814-02* (PEY) | Rao Guang-Yuan et al. | KF113709 | KF113815 | KF113762 | KJ570479 | KJ570571 |
| *Rhodiola ovatisepala* var. *ovatisepala* (Raym.-Hamet) S. H. Fu | China. Xizang: Jilong Xian | 28° 30' 59'' | 85° 13' 10'' | *G. Y. Rao et al. 100818-02* (PEY) | Rao Guang-Yuan et al. | KP114761 | KP115167 | KP114956 | - | KP115059 |
| *Rhodiola ovatisepala* var. *ovatisepala* (Raym.-Hamet) S. H. Fu | China. Xizang: Nielamu Xian | 28° 05' 31'' | 85° 57' 32'' | *G. Y. Rao et al. 100814-02-02* (PEY) | Rao Guang-Yuan et al. | KP114762 | KP115168 | KP114957 | - | KP115060 |
| *Rhodiola prainii* (Raym.-Hamet) H. Ohba | China. Xizang: Jilong Xian | 28° 30' 61'' | 85° 13' 10'' | *G. Y. Rao et al. 100818-01* (PEY) | Rao Guang-Yuan et al. | KF113711 | KF113817 | KF113764 | KJ570480 | KJ570572 |
| *Rhodiola prainii* (Raym.-Hamet) H. Ohba | China. Xizang: Jilong Xian | 28° 30' 52'' | 85° 13' 18'' | *G. Y. Rao et al. 100818-01-03* (PEY) | Rao Guang-Yuan et al. | KP114763 | KP115169 | KP114958 | KP114854 | KP115061 |
| *Rhodiola prainii* (Raym.-Hamet) H. Ohba | China. Xizang: Jilong Xian | 28° 29' 59'' | 85° 13' 12'' | *G. Y. Rao et al. 100818-01-04* (PEY) | Rao Guang-Yuan et al. | KP114764 | KP115170 | KP114959 | - | KP115062 |
| *Rhodiola prainii* (Raym.-Hamet) H. Ohba | China. Xizang: Jilong Xian | 28° 30' 62'' | 85° 13' 29'' | *G. Y. Rao et al. 100818-01-06* (PEY) | Rao Guang-Yuan et al. | KP114765 | KP115171 | KP114960 | KP114855 | KP115063 |
| *Rhodiola prainii* (Raym.-Hamet) H. Ohba | China. Xizang: Jilong Xian | 28° 30' 66'' | 85° 13' 36'' | *G. Y. Rao et al. 100818-01-05* (PEY) | Rao Guang-Yuan et al. | KP114766 | KP115172 | KP114961 | - | KP115064 |
| *Rhodiola purpureoviridis* (Praeger) S. H. Fu | China. Xizang: Linzhi Xian | 29° 33' 64'' | 94° 34' 42'' | *J. Q. Zhang et al. 120717-03-09* (PEY) | Zhang Jian-Qiang et al. | KF113712 | KF113818 | KF113765 | KJ570481 | KJ570573 |
| *Rhodiola purpureoviridis* (Praeger) S. H. Fu | China. Xizang: Linzhi Xian | 29° 33' 25'' | 94° 34' 41'' | *J. Q. Zhang et al. 120717-03-08* (PEY) | Zhang Jian-Qiang et al. | KP114767 | KP115173 | KP114962 | KP114856 | KP115065 |
| *Rhodiola purpureoviridis* (Praeger) S. H. Fu | China. Xizang: Linzhi Xian | 29° 33' 49'' | 94° 34' 51'' | *J. Q. Zhang et al. 120717-03-15* (PEY) | Zhang Jian-Qiang et al. | KP114768 | KP115174 | KP114963 | KP114857 | KP115066 |
| *Rhodiola quadrifida* (Pall.) Fisch. et Mey. | China. Qinghai: Yushu Xian | 32° 59' 68'' | 97° 10' 91'' | *G. Y. Rao et al. 100828-01* (PEY) | Rao Guang-Yuan et al. | KP114769 | KP115175 | KP114964 | KP114858 | KP115067 |
| *Rhodiola quadrifida* (Pall.) Fisch. et Mey. | China. Qinghai: Yushu Xian | 32° 51' 18'' | 97° 08' 73'' | *J. Q. Zhang et al. 100829-02* (PEY) | Zhang Jian-Qiang et al. | KF113714 | KF113820 | KF113767 | KJ570483 | KJ570575 |
| *Rhodiola quadrifida* (Pall.) Fisch. et Mey. | China. Qinghai: Yushu Xian | 32° 59' 68'' | 97° 10' 91'' | *J. Q. Zhang et al. 100828-01-1c* (PEY) | Rao Guang-Yuan et al. | KP114770 | KP115176 | KP114965 | - | KP115068 |
| *Rhodiola quadrifida* (Pall.) Fisch. et Mey. | China. Qinghai: Yushu Xian | 32° 51' 18'' | 97° 08' 73'' | *J. Q. Zhang et al. 100829-02-2c* (PEY) | Zhang Jian-Qiang et al. | KP114771 | KP115177 | KP114966 | - | KP115069 |
| *Rhodiola rhodantha* (A. Gray) H. Jacobsen | USA. Colorado | 37° 55' 43'' | -107° 30' 52'' | *H. Guest 6039* (UVIC) | Heidi Guest | KJ569943 | KJ570311 | KJ570073 | KJ570484 | KJ570576 |
| *Rhodiola rhodantha* (A. Gray) H. Jacobsen | USA. Colorado | 37° 05' 23'' | -107° 29' 46'' | *J. Ackerfield 3389* (CS) | Jennifer Ackerfield | KJ569944 | - | KJ570074 | KJ570485 | - |
| *Rhodiola rhodantha* (A. Gray) H. Jacobsen | USA. Colorado | 38° 02' 41'' | -107° 25' 51'' | *E. Hott 2664* (CS) | Emily Hott | KJ569945 | KJ570312 | KJ570075 | KJ570486 | KJ570577 |
| *Rhodiola rhodantha* (A. Gray) H. Jacobsen | USA. Uhta | 40° 42' 28'' | -110° 52' 07'' | *G. Allen 1335* (UVIC) | Geraldine Allen | - | KJ570314 | KJ570077 | KJ570488 | - |
| *Rhodiola rhodantha* (A. Gray) H. Jacobsen | USA. Wyoming | 41° 21' 35'' | -106° 18' 36'' | *H. Guest 6026* (UVIC) | Heidi Guest | KF113715 | KF113821 | KF113768 | KJ570489 | - |
| *Rhodiola rosea* L. | China. Beijing: Dongling Mt. | 39° 59' 21'' | 115° 25' 38'' | *J. Q. Zhang 100717-01* (PEY) | Zhang Jian-Qiang et al. | KF113717 | KF113823 | KJ570083 | KJ570495 | KJ570582 |
| *Rhodiola rosea* L. | China. Beijing: Dongling Mt. | 39° 59' 27'' | 115° 25' 36'' | *J. Q. Zhang 100717-02* (PEY) | Zhang Jian-Qiang et al. | KP114772 | KP115178 | KP114967 | KP114859 | KP115070 |
| *Rhodiola rosea* L. | Canada. New Brunswick: Cap Enragé | 45° 35' 38'' | -64° 46' 48'' | *RR2006-NB-cap* (MT) | Mariannick Archambault | KJ569947 | KJ570315 | KJ570078 | KJ570490 | KJ570579 |
| *Rhodiola rosea* L. | Russia. Chukotka | 66° 48' 59'' | 176° 11' 08'' | *H. Solsad & R. Elven 05/0863* (ALA) | H. Solstad & R. Elven | KJ569948 | KJ570316 | KJ570079 | KJ570491 | - |
| *Rhodiola rosea* L. | Greenland | 65° 02' 23'' | -52° 26' 56'' | *R. W. Bartlett 474* (US) | Rupert W. Bartlett | - | KJ570318 | KJ570081 | KJ570493 | KJ570580 |
| *Rhodiola rosea* L. | Iceland | 64° 21' 04'' | -18° 08' 36'' | *M. Hauksdottir s.n.* (US) | Margret Hauksdottir | KJ569949 | KJ570319 | KJ570082 | KJ570494 | KJ570581 |
| *Rhodiola rosea* L. | China. Beijing: Wuling Mt. | 40° 35' 96'' | 117° 29' 11'' | *J. Q. Zhang et al. 120616-02* (PEY) | Zhang Jian-Qiang et al. | KJ569950 | KJ570320 | KJ570084 | KJ570496 | KJ570583 |
| *Rhodiola rosea* L. | China. Beijing: Wutai Mt. | 39° 03' 56'' | 113° 38' 56'' | *J. Q. Zhang et al. 120815-03* (PEY) | Zhang Jian-Qiang et al. | KJ569951 | KJ570321 | KJ570085 | KJ570497 | KJ570584 |
| *Rhodiola sachalinensis* A. Bor. | China. Jilin: Changbai Mt. | 42° 11' 10'' | 128° 10' 41'' | *J. Q. Zhang 110911-02* (PEY) | Zhang Jian-Qiang et al. | KF113718 | KF113824 | KF113771 | KJ570498 | KJ570585 |
| *Rhodiola sacra* var. *sacra* (Prain ex Raym.-Hamet) S. H. Fu | China. Xizang: Langkazi Xian | 29° 01' 22'' | 87° 30' 28'' | *G. Y. Rao 090731-03* (PEY) | Rao Guang-Yuan | KP114773 | KP115179 | KP114968 | KP114860 | KP115071 |
| *Rhodiola sacra* var. *sacra* (Prain ex Raym.-Hamet) S. H. Fu | China. Xizang: Namulin Xian | 30° 04' 48'' | 89° 06' 25'' | *G. Y. Rao et al. 100811-01* (PEY) | Rao Guang-Yuan et al. | KF113719 | KF113825 | KF113772 | KJ570499 | KJ570586 |
| *Rhodiola sacra* var. *sacra* (Prain ex Raym.-Hamet) S. H. Fu | China. Xizang: Qushui Xian | 29° 37' 75'' | 91° 11' 10'' | *G. Y. Rao et al. 100823-05* (PEY) | Rao Guang-Yuan et al. | KP114774 | KP115180 | KP114969 | KP114861 | KP115072 |
| *Rhodiola sacra* var. *sacra* (Prain ex Raym.-Hamet) S. H. Fu | China. Xizang: Qushui Xian | 29° 21' 08'' | 90° 43' 94'' | *G. Y. Rao et al. 100823-03* (PEY) | Rao Guang-Yuan et al. | KP114775 | KP115181 | KP114970 | KP114862 | KP115073 |
| *Rhodiola sacra* var. *sacra* (Prain ex Raym.-Hamet) S. H. Fu | China. Xizang | 29° 42' 51'' | 91° 06' 23'' | *Tibet-MacArthur 145* (US) | Wen Jun et al. | - | KJ570322 | KJ570086 | KJ570500 | KJ570587 |
| *Rhodiola serrata* H. Ohba | China. Xizang: Milin Xian | 29° 06' 96'' | 93° 52' 29'' | *J. Q. Zhang et al. 120719-04-10* (PEY) | Zhang Jian-Qiang et al. | KF113721 | KF113827 | KF113774 | KJ570503 | KJ570590 |
| *Rhodiola serrata* H. Ohba | China. Xizang: Milin Xian | 29° 06' 49'' | 93° 52' 10'' | *J. Q. Zhang et al. 120719-04-05* (PEY) | Zhang Jian-Qiang et al. | KP114776 | KP115182 | KP114971 | KP114863 | KP115074 |
| *Rhodiola serrata* H. Ohba | China. Xizang: Milin Xian | 29° 06' 15'' | 93° 52' 14'' | *J. Q. Zhang et al. 120719-04-03* (PEY) | Zhang Jian-Qiang et al. | KP114777 | KP115183 | KP114972 | KP114864 | KP115075 |
| *Rhodiola sexifolia* S. H. Fu | China. Sichuan: Muli Xian | 28° 07' 42'' | 101° 07' 79'' | *J. Q. Zhang et al. 110818-01* (PEY) | Zhang Jian-Qiang et al. | KF113722 | KF113828 | KF113775 | KJ570504 | KJ570591 |
| *Rhodiola sexifolia* S. H. Fu | China. Xizang: Chaya Xian | 30° 40' 56'' | 97° 15' 08'' | *G. Y. Rao 090725-02* (PEY) | Rao Guang-Yuan | KP114778 | KP115184 | KP114973 | KP114865 | KP115076 |
| *Rhodiola sexifolia* S. H. Fu | China. Xizang: Qushui Xian | 29° 21' 08'' | 90° 43' 94'' | *G. Y. Rao et al. 100823-01* (PEY) | Rao Guang-Yuan et al. | KP114779 | KP115185 | KP114974 | KP114866 | KP115077 |
| *Rhodiola sexifolia* S. H. Fu | China. Xizang: Qushui Xian | 29° 21' 09'' | 90° 43' 52'' | *G. Y. Rao et al. 100823-01-03* (PEY) | Rao Guang-Yuan et al. | KP114780 | KP115186 | KP114975 | KP114867 | KP115078 |
| *Rhodiola sexifolia* S. H. Fu | China. Xizang | 30° 02' 24'' | 93° 57' 02'' | *Tibet-MacArthur 3135* (US) | Wen Jun et al. | - | KJ570324 | KJ570088 | KJ570505 | KJ570592 |
| *Rhodiola sinuata* (Royle ex Edgew.) S .H. Fu | [China. Yuannan: Shangri-La Xian](app:ds:Shangri-La) | 27° 47' 61'' | 99° 48' 76'' | *J. Q. Zhang et al. 110801-02-09* (PEY) | Zhang Jian-Qiang et al. | KF113723 | KF113829 | KF113776 | KJ570506 | KJ570593 |
| *Rhodiola sinuata* (Royle ex Edgew.) S .H. Fu | [China. Yuannan: Shangri-La Xian](app:ds:Shangri-La) | 27° 47' 66'' | 99° 48' 72'' | *J. Q. Zhang et al. 110801-02-05* (PEY) | Zhang Jian-Qiang et al. | KP114781 | KP115187 | KP114976 | KP114868 | KP115079 |
| *Rhodiola sinuata* (Royle ex Edgew.) S .H. Fu | [China. Yuannan: Shangri-La Xian](app:ds:Shangri-La) | 27° 47' 63'' | 99° 48' 79'' | *J. Q. Zhang et al. 110801-02-04* (PEY) | Zhang Jian-Qiang et al. | KP114782 | KP115188 | KP114977 | KP114869 | KP115080 |
| *Rhodiola sinuata* (Royle ex Edgew.) S .H. Fu | [China. Yuannan: Shangri-La Xian](app:ds:Shangri-La) | 27° 47' 50'' | 99° 48' 54'' | *J. Q. Zhang et al. 110801-02-01* (PEY) | Zhang Jian-Qiang et al. | KP114783 | KP115189 | KP114978 | KP114870 | KP115081 |
| *Rhodiola sinuata* (Royle ex Edgew.) S .H. Fu | [China. Yuannan: Shangri-La Xian](app:ds:Shangri-La) | 27° 47' 48'' | 99° 48' 96'' | *J. Q. Zhang et al. 110801-02-10* (PEY) | Zhang Jian-Qiang et al. | KP114784 | KP115190 | KP114979 | KP114871 | KP115082 |
| *Rhodiola smithii* (Raym.-Hamet) S. H. Fu | China. Xizang: Nanmulin Xian | 29° 41' 91'' | 89° 05' 28'' | *G. Y. Rao et al. 100810-02* (PEY) | Rao Guang-Yuan et al. | KF113724 | KF113830 | KF113777 | KJ570507 | KJ570594 |
| *Rhodiola smithii* (Raym.-Hamet) S. H. Fu | China. Xizang: Jilong Xian | 28° 53' 72'' | 85° 22' 52'' | *G. Y. Rao et al. 100819-02* (PEY) | Rao Guang-Yuan et al. | KP114785 | KP115191 | KP114980 | KP114872 | KP115083 |
| *Rhodiola smithii* (Raym.-Hamet) S. H. Fu | China. Xizang: Nanmulin Xian | 29° 41' 91'' | 89° 05' 28'' | *G. Y. Rao et al. 100810-02-06* (PEY) | Rao Guang-Yuan et al. | KP114786 | KP115192 | KP114981 | KP114873 | KP115084 |
| *Rhodiola smithii* (Raym.-Hamet) S. H. Fu | China. Xizang: Nanmulin Xian | 29° 41' 91'' | 89° 05' 28'' | *G. Y. Rao et al. 100810-02-04* (PEY) | Rao Guang-Yuan et al. | KP114787 | KP115193 | KP114982 | KP114874 | KP115085 |
| *Rhodiola smithii* (Raym.-Hamet) S. H. Fu | China. Xizang: Jilong Xian | 28° 53' 72'' | 85° 22' 52'' | *G. Y. Rao et al. 100819-02-02* (PEY) | Rao Guang-Yuan et al. | KP114788 | KP115194 | KP114983 | KP114875 | KP115086 |
| *Rhodiola stapfii* (Raym.-Hamet) S. H. Fu | China. Xizang: Qusong Xian | 29° 01' 21'' | 92° 21' 49'' | *J. Q. Zhang 120722-01-07* (PEY) | Zhang Jian-Qiang | KF113726 | KF113832 | KF113779 | KJ570508 | KJ570595 |
| *Rhodiola stapfii* (Raym.-Hamet) S. H. Fu | China. Xizang: Qusong Xian | 29° 01' 28'' | 92° 21' 48'' | *J. Q. Zhang 120722-01-02* (PEY) | Zhang Jian-Qiang | KP114789 | KP115195 | KP114984 | KP114876 | KP115087 |
| *Rhodiola stapfii* (Raym.-Hamet) S. H. Fu | China. Xizang: Qusong Xian | 29° 01' 25'' | 92° 21' 59'' | *J. Q. Zhang 120722-01-01* (PEY) | Zhang Jian-Qiang | KP114790 | KP115196 | KP114985 | KP114877 | KP115088 |
| *Rhodiola tangutica* (Maxim.) S. H. Fu | China. Qinghai: Kunlun Mt. | 35° 38' 55'' | 94° 04' 35'' | *G. Y. Rao et al. 100807-01* (PEY) | Rao Guang-Yuan et al. | KF113727 | KF113833 | KF113780 | KJ570509 | KJ570596 |
| *Rhodiola tangutica* (Maxim.) S. H. Fu | China. Qinghai: Kunlun Mt. | 35° 38' 55'' | 94° 04' 35'' | *G. Y. Rao et al. 100807-02* (PEY) | Rao Guang-Yuan et al. | KP114791 | KP115197 | KP114986 | KP114878 | KP115089 |
| *Rhodiola tangutica* (Maxim.) S. H. Fu | China. Qinghai: Kunlun Mt. | 35° 38' 55'' | 94° 04' 35'' | *G. Y. Rao et al. 100807-01-4c* (PEY) | Rao Guang-Yuan et al. | KP114792 | KP115198 | KP114987 | KP114879 | KP115090 |
| *Rhodiola tangutica* (Maxim.) S. H. Fu | China. Qinghai: Kunlun Mt. | 35° 38' 55'' | 94° 04' 35'' | *G. Y. Rao et al. 100807-02-7x* (PEY) | Rao Guang-Yuan et al. | KP114793 | KP115199 | KP114988 | KP114880 | KP115091 |
| *Rhodiola tangutica* (Maxim.) S. H. Fu | China. Qinghai: Kunlun Mt. | 35° 38' 55'' | 94° 04' 35'' | *G. Y. Rao et al. 100807-02-10x* (PEY) | Rao Guang-Yuan et al. | KP114794 | KP115200 | KP114989 | KP114881 | KP115092 |
| *Rhodiola tibetica* (Hook. f. & Thomson) S. H. Fu | China. Xizang: Nanmulin Xian | 29° 50' 18'' | 92° 19' 26'' | *G. Y. Rao 090728-03* (PEY) | Rao Guang-Yuan | KP114795 | KP115201 | KP114990 | KP114882 | KP115093 |
| *Rhodiola tibetica* (Hook. f. & Thomson) S. H. Fu | China. Xizang: Nanmulin Xian | 30° 04' 46'' | 89° 06' 24'' | *G. Y. Rao et al. 100811-03-01* (PEY) | Rao Guang-Yuan et al. | KF113728 | KF113834 | KF113781 | KJ570510 | KJ570597 |
| *Rhodiola tibetica* (Hook. f. & Thomson) S. H. Fu | China. Xizang: Nanmulin Xian | 30° 04' 48'' | 89° 06' 23'' | *G. Y. Rao et al. 100811-05* (PEY) | Rao Guang-Yuan et al. | KP114796 | KP115202 | KP114991 | KP114883 | KP115094 |
| *Rhodiola tibetica* (Hook. f. & Thomson) S. H. Fu | China. Xizang: Nanmulin Xian | 30° 04' 49'' | 89° 06' 01'' | *G. Y. Rao et al. 100811-05-03* (PEY) | Rao Guang-Yuan et al. | KP114797 | KP115203 | KP114992 | KP114884 | KP115095 |
| *Rhodiola tibetica* (Hook. f. & Thomson) S. H. Fu | China. Xizang | 30° 02' 56'' | 94° 12' 26'' | *Tibet-MacArthur 3235* (US) | Wen Jun et al. | - | KJ570325 | KJ570089 | KJ570511 | KJ570598 |
| *Rhodiola wallichiana* (Hook.) S. H. Fu | China. Xizang: Nielamu Xian | 28° 07' 26'' | 85° 57' 43'' | *G. Y. Rao et al. 100815-02* (PEY) | Rao Guang-Yuan et al. | KF113730 | KF113836 | KF113783 | KJ570512 | KJ570599 |
| *Rhodiola wallichiana* (Hook.) S. H. Fu | China. Xizang: Nielamu Xian | 28° 07' 26'' | 85° 57' 43'' | *G. Y. Rao et al. 100815-02-01* (PEY) | Rao Guang-Yuan et al. | KP114798 | KP115204 | KP114993 | KP114885 | KP115096 |
| *Rhodiola wallichiana* (Hook.) S. H. Fu | China. Xizang: Nielamu Xian | 28° 07' 26'' | 85° 57' 43'' | *G. Y. Rao et al. 100815-02-03* (PEY) | Rao Guang-Yuan et al. | KP114799 | KP115205 | KP114994 | KP114886 | KP115097 |
| *Rhodiola wallichiana* (Hook.) S. H. Fu | China. Xizang | 28° 07' 25'' | 85° 59' 32'' | *Tibet-MacArthur 472* (US) | Wen Jun et al. | KJ569953 | KJ570326 | KJ570090 | KJ570513 | KJ570600 |
| *Rhodiola wallichiana* (Hook.) S. H. Fu | China. Xizang | 28° 05' 01'' | 86° 01' 20'' | *Tibet-MacArthur 775* (US) | Wen Jun et al. | KJ569954 | KJ570327 | KJ570091 | KJ570514 | KJ570601 |
| *Rhodiola yunnanensis* (Franch.) S. H. Fu | [China. Yunnan: Shangri-La Xian](app:ds:Shangri-La) | 27° 53' 54'' | 99° 33' 42'' | *J. Q. Zhang et al. 110730-01* (PEY) | Zhang Jian-Qiang et al. | KF113731 | KF113837 | KF113784 | KJ570515 | KJ570602 |
| *Rhodiola yunnanensis* (Franch.) S. H. Fu | China. Yunnan: Yulong Mt. | 27° 02' 79'' | 100° 11' 67'' | *J. Q. Zhang et al. 110728-02-01* (PEY) | Zhang Jian-Qiang et al. | KP114800 | KP115206 | KP114995 | KP114887 | KP115098 |
| *Rhodiola yunnanensis* (Franch.) S. H. Fu | China. Yunnan: Yulong Mt. | 27° 02' 79'' | 100° 11' 67'' | *J. Q. Zhang et al. 110728-02-02* (PEY) | Zhang Jian-Qiang et al. | KP114801 | KP115207 | KP114996 | KP114888 | KP115099 |
| *Rhodiola yunnanensis* (Franch.) S. H. Fu | China. Yunnan: Shangrila | 27° 57' 21'' | 99° 35' 26'' | *Tibet-MacArthur 2345* (US) | Wen Jun et al. | KJ569955 | KJ570328 | KJ570092 | KJ570516 | KJ570603 |
| *Rhodiola yunnanensis* (Franch.) S. H. Fu | China. Yunnan: Shangrila | 27° 54' 59'' | 99° 29' 51'' | *Tibet-MacArthur 1219* (US) | Wen Jun et al. | KJ569956 | KJ570329 | KJ570093 | KJ570517 | KJ570604 |
